# Effects of Poncirin, a Citrus Flavonoid and Its Aglycone, Isosakuranetin, on the Gut Microbial Diversity and Metabolomics in Mice

**DOI:** 10.3390/molecules27113641

**Published:** 2022-06-06

**Authors:** Xuedan Cao, Xiao Guo, Xiugui Fang, Shuijiang Ru, Erhu Li

**Affiliations:** 1 Zhejiang Citrus Research Institute, Taizhou 318026, China; fangxg640103@163.com (X.F.); rushj@163.com (S.R.); 2 College of Food Science and Technology, Huazhong Agricultural University, Wuhan 430070, China; 15871700925@163.com (X.G.); erhuli@mail.hzau.edu.cn (E.L.)

**Keywords:** poncirin, isosakuranetin, gut microbiota, SCFAs, urine metabolomics

## Abstract

Poncirin (PC) and its aglycone, isosakuranetin (IR), occur naturally in citrus fruits. This study aimed to explore the pathways behind the different health benefits of PC and IR by evaluating the effect of these two bioactive flavonoids on the gut microbial diversity and metabolomics of mice. The 16S rRNA gene sequencing was used to analyze the alteration of gut microbiota in mice after PC and IR intervention. The metabolic impact of PC and IR in mice were studied using a metabolomics approach based on LC-MS analysis. Results showed that, after 7 days intervention, PC and IR multiplied the abundance of *Parabacteroides* in mice’s intestinal tracts by 1.2 and 1.0 times, respectively. PC increased the abundance of *Bacteroides* by 2.4 times. IR reduced the *Allobaculum* abundance by 1.0 time and increased *Alloprevotella* abundance by 1.5 times. When mice were given PC, their fecal acetic acid level increased by 1.8 times, while their isobutyric and isovaleric acid content increased by 1.2 and 1.3 times, respectively. Supplementation with IR had no significant effect on the content of short-chain fatty acids (SCFAs) in the feces of mice. The potential urine biomarkers of mice in the PC group were involved in the digestion and absorption of protein and carbohydrate, as well as the metabolism of amino acids, such as glycine, serine, threonine, tryptophan, D-arginine, D-ornithine, etc. IR mainly affected the amino acid metabolic pathways in mice, including taurine and hypotaurine metabolism, glutathione metabolism, histidine metabolism, D-glutamate metabolism, etc. This study provided valuable clues for future research on the health promoting mechanisms of PC and IR.

## 1. Introduction

**Figure 1:**
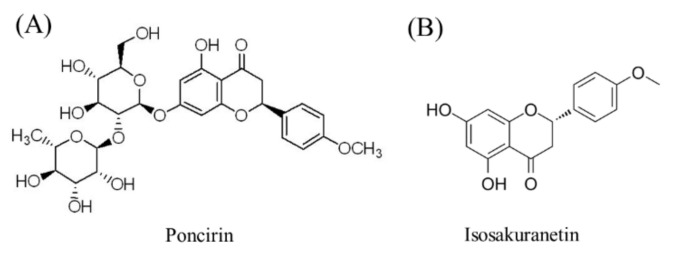
Chemical structure of poncirin (**A**) and isosakuranetin (**B**).

Poncirin (Figure 1A) is a natural flavanone glycoside found in many citrus fruit species [1,2], and has many health benefits, including antioxidation, anti-inflammatory and immunoregulatory [3,4]. A study revealed that poncirin exhibits excellent antioxidant activity due to its potent scavenging activity against DPPH free radicals [5]. In a liver injury mice model, poncirin treatment significantly improved the hematological and biochemical parameters, along with induced anti-oxidants and reduced inflammatory mediators [4]. Moreover, poncirin reportedly attenuates anti-Alzheimer’s disease activity through acetylcholinesterase inhibition [6], and acetylcholinesterase inhibition can ameliorate brain ischemia–reperfusion injury. Additionally, poncirin might be useful for the treatment of diabetes mellitus, a chronic metabolic disease, and its related complications [7].

Isosakuranetin (Figure 1B) is an aglycone form of poncirin. It is well known that there is an apparent difference in the bioavailability of flavonoids and their aglycones [8], which leads to some differences in their health benefits. Isosakuranetin is found in citrus fruits, and can be produced by the transformation of poncirin by microorganisms. Our previous study shown that the poncirin in citrus juice was transformed into isosakuranetin after fermentation by *Lactobacillus paracei* LPC37 [9]. The fermented citrus juice had more effective anti-obesity activity than unfermented juice. Obesity is also closely related to metabolic syndromes, such as hyperlipidemia and hyperglycemia. At present, it is still unclear how poncirin and its aglycone isosakuranetin lead to the functional difference between fermented and unfermented citrus juice.

Phytochemicals perform their biological functions at different body levels in the form of metabolites [10]. After oral administration, phytochemicals are preliminarily enzymatically hydrolyzed in the epithelium of the oral cavity or the epithelial cells of the small intestine, and also by the intestinal microflora, to form the hydroxylated, hydrogenated, dehydrogenated, and acetylated metabolites [11]. Subsequently, the metabolites are transported, mainly via the portal vein, to the liver for further biotransformation to glucuronide and sulphate conjugates through phase I and phase II metabolic reactions [12]. Ultimately, the metabolites and unabsorbed chemicals are further metabolized by microbes to yield low molecular weight catabolites, which have been identified by metabolomics in urine and fecal samples [13,14]. Metabolomics analysis of phytochemicals can reveal the bioavailable metabolites to elucidate the health benefits of phytochemicals.

The gut microbiota is a critical mediator in maintaining health [15]. The gut microbiota interacts with various organs and systems in the body, including brain, lung, liver, bone, cardiovascular system, among others. Moreover, the microbiota-derived metabolites, such as short chain fatty acids (SCFA), regulate physiological homeostasis [16]. Meanwhile, physiological homeostasis may be disrupted by the microbiota, resulting in disruption of the host metabolism, immune dysregulation, neurological and cognitive dysfunction, and other conditions [17]. This disrupted status would cause a series of disorders, including obesity, diabetes, autoimmunity, allergy, inflammatory bowel disease (IBD), and cancer [18]. Desirable modulation of gut bacteria by phytochemicals can confer health benefits, including increased abundance of probiotic gut microbiota [19], reduced abundance of pathogenic bacteria [20], and activation of SCFA excretion [21].

The aim of this study was to explore the metabolomics of poncirin and its aglycone in mice and their short-term effects on gut microbiota of healthy mice, so as to elucidate the pathways behind the different biological effects of poncirin and its aglycone. This study will provide a reference for the exploration of the functions and mechanisms of fermented citrus products, which are rich in a variety of flavonoids and aglycones.

## 2. Results

### 2.1. Gut Microbiota Diversity Variation after PC or IR Supplementation

The overall structural changes of gut microbiota in response to PC or IR administration were determined by analysis of the 16S rRNA gene sequences of microbial samples isolated from the feces of Control, PC, and IR mouse groups. UniFrac distance-based principal component analysis (PCA) revealed distinct clustering of intestinal microbe communities for each experimental group.

**Figure 2:**
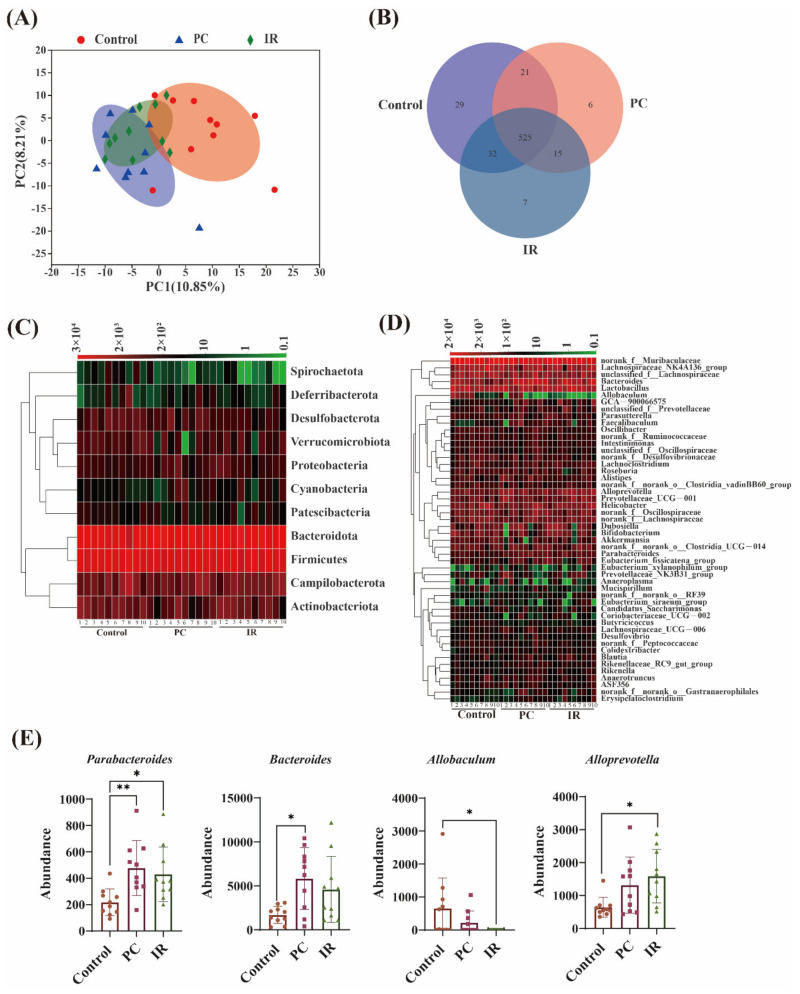
PC and IR alter the composition of gut microbiota in mice. (**A**) PCA clustering analysis. (**B**) Venn diagrams showing the unique and shared OTUs in the gut microbiota among groups. (**C**) Heatmap indicating the relative contribution of the top 11 dominant phylum and (**D**) top 50 dominant genera in each group. (**E**) The gut microbiota with significant differences in abundance among groups. One-way ANOVA was used to analyze statistical differences, * *p* < 0.05, ** *p* < 0.01.

Remarkable changes in the microbiota community structure were induced by both PC and IR intervention. The microbes in the IR and control groups were more closely clustered in comparison with the PC and control groups, which indicates that PC consumption induced more significant microbial composition changes (Figure 2A). Venn diagram analysis indicated the shared richness of gut microbiota in different groups (Figure 2B). Furthermore, we compared the relative abundance of the predominant taxa in the three groups. At the phylum level, all fecal samples shared similar community structures. *Firmicutes* and *Bacteroidetes* were the dominant phyla, representing more than 80% of the relative abundance (Figure 2C). The relative abundance of *Spirochaetota*, *Actinobacteriota*, *Desulfobacterota*, *Verrucomicrobiota*, and *Deferribacterota* were decreased in the PC or IR-treated mice (Figure 2C). The relative abundance of *Proteobacteria* and *Cyanobacteria* were increased in the mice after PC or IR treatment (Figure 2C). At the genus level, we observed that the relative abundance of *Alloprevotella*, *Dubosiella*, *Prevotellaceae_UCG-001*, *Bacteroides*, and *Parabacteroides* were increased after PC or IR supplementation (Figure 2D).

Figure 2E presents the microbiota with significant differences. IR treatment significantly boosted *Parabacteroides* and *Alloprevotella* abundance and significantly decreased *Allobaculum* abundance. PC treatment significantly increased *Parabacteroides* and *Bacteroides* abundance.

### 2.2. SCFA Concentrations Variation in Feces after PC or IR Supplementation

**Figure 3:**
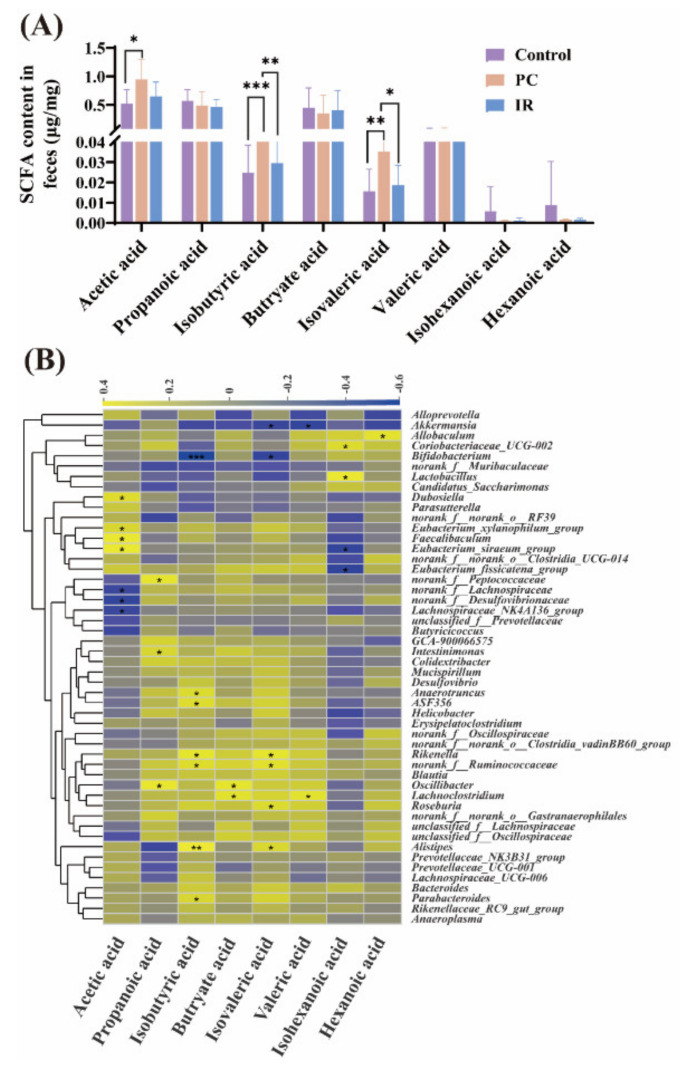
Effects of PC or IR treatment on the feces at the metabolite level. (**A**) The SCFA levels. (**B**) Pearson correlation analysis was performed between various fecal metabolites and gut microbiota. The color scale represents the strength of correlation, ranging from 0.4 (strong positive correlation) to 0.6 (strong negative correlation). One-way ANOVA was used to analyze statistical differences; * *p* < 0.05, ** *p* < 0.01, and *** *p* < 0.001.

The dominated SCFA in feces in different groups are acetic, propionic, and butyric acids, which account for over 90% of the total SCFA (Figure 3A). The contents of acetic, isobutyric, and isovaleric acids in the feces of the PC group were significantly higher than that of the control group (Figure 3A), while IR treatment had no distinct effect on the content of SCFA in feces (Figure 3A).

The results of correlation analysis (Figure 3B) revealed there was a significant positive correlation between acetic acid levels and *Dubosiella*, *Eubacterium_xylanophilum_group*, *Faecalibaculum*, and *Eubacterium_siraeum_group*. The propanoic acid levels were positively correlated with *Peptococcaceae*, *Intestinimonas*, and *Oscillibacter*. The butryate acid levels were positively correlated with *Oscillibacter* and *Lachnoclostridium*. The hexanoic acid levels were positively correlated with *Allobaculum*. The isobutyric acid and isovaleric acid levels were correlated negatively with *Bifidobacterium*, while correlated positively with *Rikenella*, *norank_f_Ruminococcaceae*, and *Alistipes*. The valeric acid levels were correlated negatively with *Akkermansia*, and correlated positively with *Lachnoclostridium*. The isohexanoic acid levels were significantly negatively correlated with *Eubacterium_siraeum_group* and *Eubacterium_fissicatena_group*.

### 2.3. Metabolomic Variation after PC or IR Supplementation

**Figure 4:**
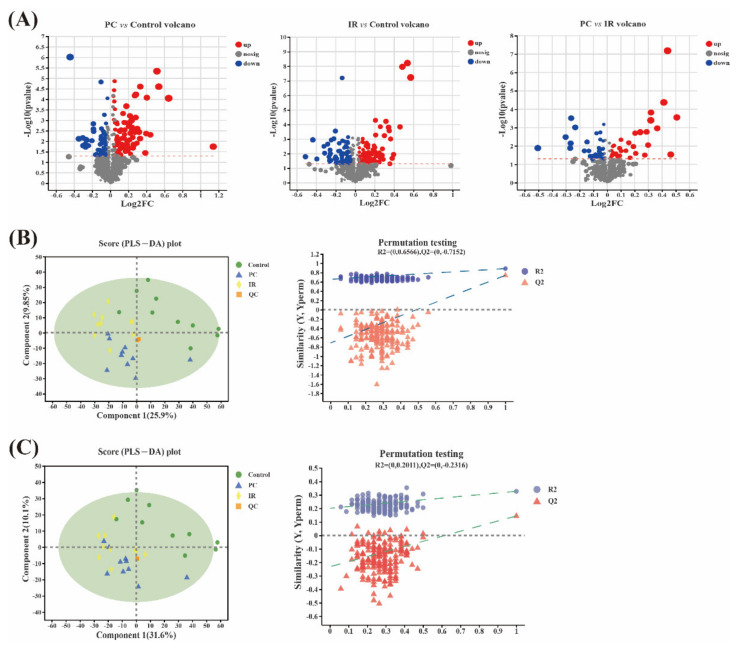
Metabolomic variation after PC or IR supplementation. (**A**) Volcano plot showing 144 variables that differ significantly between PC group and control group, 175 variables that differ significantly between IR group and control group, and 69 variables that differ significantly between PC group and IR group (red and blue dots). FC: fold change. (**B**) In positive ion mode, PLS-DA score plot of these different variables showing that they can clearly separate control group and PC/IR group, as well as PC group and IR group. QC: quality control samples. Permutation test conducted to validate the model. R2(Y) (model fit) and Q2(Y) (predictive ability) were employed to evaluate the validity and robustness of the model. (**C**) In negative ion mode, PLS-DA score plot of these different variables showing that they can clearly separate control group and PC/IR group, and that no differences between PC group and IR group occur. Permutation test conducted to validate the model. R2(Y) (model fit) and Q2(Y) (predictive ability) were employed to evaluate the validity and robustness of the model.

A volcano plot analysis was performed to visualize the metabolomic variables able to differentiate between the control group and the PC/IR group. As can be seen from Figure 4A, the volcano plot showed 144 variables that differed significantly (*t*-test *p*-value < 0.05 and abs (log2 FC) > 1) between the PC group and the control group (Supplementary Table S1), 175 variables that differed significantly between the IR group and the control group (Supplementary Table S2), and 69 variables that differed significantly between the PC group and the IR group (Supplementary Table S3). Next, a supervised Pareto scaling PLS-DA analysis was performed with these selected variables to evaluate whether they could differentiate the experimental groups and the control group, thus revealing a clear separation between the control group and the PC/IR group, as well as between the PC group and the IR group in the positive ion mode (Figure 4B). In the negative ion mode, a clear separation was observed between the control group and the PC/IR group, but no differences between the PC group and the IR group occurred (Figure 4C). Although the intercept of the Q2 regression line and the Y-axis was greater than 0.05, with the decrease in replacement retention, the regression line of R2 and Q2 showed an upward trend, indicating that there was no over-fitting phenomenon in the model (Figure 4B,C).

### 2.4. Altered Metabolic Pathways after PC or IR Supplementation

**Figure 5:**
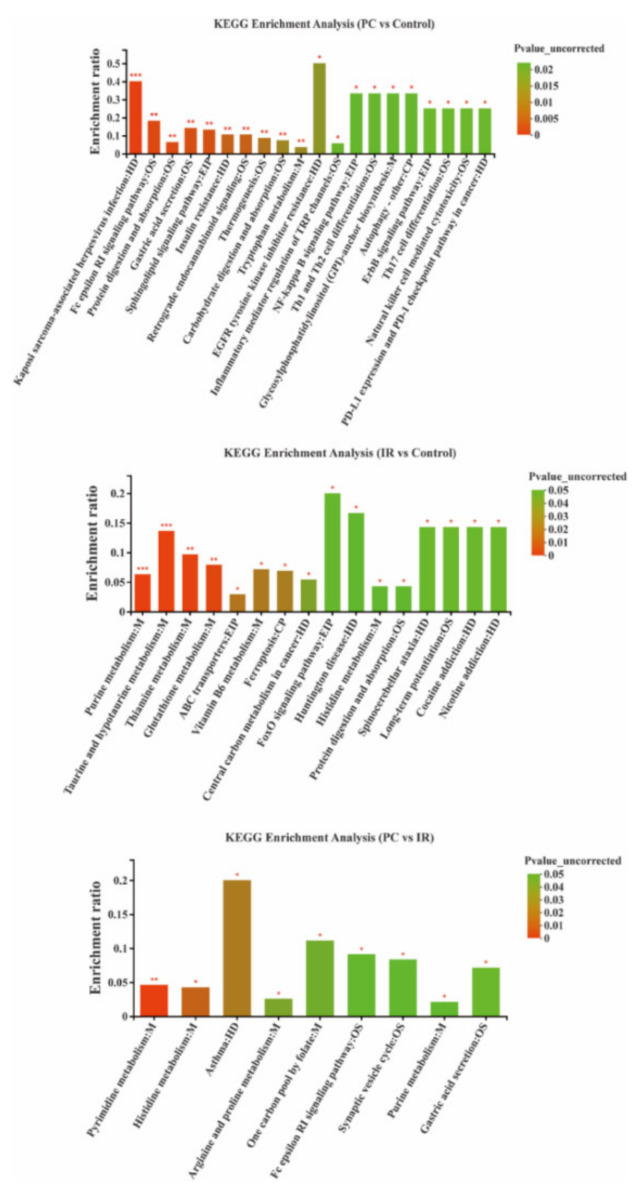
Metabolic pathway enrichment study of differentially presented metabolites between PC and control groups, IR and control groups, and PC and IR groups. CP, EIP, HD, M, and OS are the class names of the metabolic pathways in the KEGG annotation. CP: cellular processes; EIP: environmental information processing; HD: human diseases; M: metabolism; OS: organismal systems. * *p* < 0.05, ** *p* < 0.01, and *** *p* < 0.001.

The metabolite pathways enrichment analysis (Figure 5) showed that some metabolic pathways, including protein digestion and absorption, gastric acid secretion, sphingolipid signaling pathway, insulin resistance, thermogenesis, carbohydrate digestion and absorption, and tryptophan metabolism were significantly affected (*p* < 0.01) by PC. Some metabolic pathways, including purine metabolism, taurine and hypotaurine metabolism, thiamine metabolism, and glutathione metabolism were significantly affected (*p* < 0.01) by IR. Comparing the PC group with the IR group, the pyrimidine metabolism pathway was significantly changed (*p* < 0.01). Most of these pathways were involved in metabolism and human disease.

**Figure 6:**
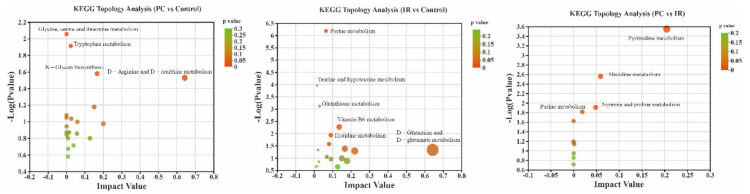
Topology analysis of differentially presented metabolites between PC and control groups, IR and control groups, and PC and IR groups. The X-axis represents the pathway impact, and Y-axis represents the pathway enrichment. Larger sizes and darker colors represent greater pathway enrichment and higher pathway impact values, respectively.

Pathway enrichment and pathway topology analysis were performed using MetaboAnalyst 4.0, which is based on high-quality KEGG metabolic pathways as the backend knowledgebase (Figure 6). Results indicated that four metabolic pathways that showed significant responses to PC treatment included glycine, serine and threonine metabolism, tryplophan metabolism, N-glycan biosynthesis, and D-arginine and D-ornithine metabolism. The six metabolic pathways that showed significant responses to IR treatment included purine metabolism, taurine and hypotaurine metabolism, glutathione metabolism, vitamin B6 metabolism, histidine metabolism, and D-glutamine and D-glutamate metabolism. Three perturbed metabolic pathways showed lower *p*-values and higher pathway impact between the PC and IR groups; these included pathways related to pyrimidine metabolism, histidine metabolism, arginine and proline metabolism, and purine metabolism.

## 3. Discussion

Poncirin (PC) and isosakuranetin (IR) are widely found in citrus fruits (in particular, oranges, mandarins, tangors, grapefruits, tangelos, and chinotto) and fermented citrus products, however, their content in citrus fruits or fermented citrus products is much lower than that of hesperidin and naringin, which has led to their beneficial effects not being fully studied in recent years. Further studies are required to clarify the mechanisms underlining their health-promoting properties.

PC and IR increased the number of *Proteobacteria*, *Cyanobacteria*, *Alloprevotella*, *Dubosiella*, *Prevotellaceae_UCG-001*, *Bacteroides*, and *Parabacteroides* in mice. *Bacteroides*, Pre*votellaceae_UCG-001*, and *Parabacteroides* belong to intestinal mucosa-associated microbiota [22]. In humans, there is a stable dominant mucosa-associated microbiota from the ileum to the rectum. According to a previous study, the majority of these microbes were symbiotic or mutualistic to the host, providing a line of defense against pathogens, modulating the immune system, processing nutrients, and stimulating various host activities [23].

*Alloprevotella* can produce short-chain fatty acids (SCFA), and have a capacity for providing energy for intestinal cells and protecting the gut barrier [24]. Wan et al. [25] found that the control group contained more *Dubosiella* than the dextran sulphate sodium (DSS)-induced colitis mice, indicating that *Dubosiella* may be potentially beneficial bacteria to fight against colitis. Previous study has shown that *Proteobacteria* and *Actinobacteria* were representative of a cellulolytic bacterial community in the intestine, and played important roles in digesting plant material and providing energy and nutrients for the host [26].

However, *Cyanobacteria* may be a maleficent bacterium. It has been reported to have had a positive correlation to the LDL-C level, which could induce fat accumulation and obesity in subjects with primary hyperlipidemia [27].

The numbers of *Actinobacteriota*, *Deferribacterota, Spirochaetota*, *Desulfobacterota*, and *Verrucomicrobiota* were decreased after PC or IR administration. It has been reported that decreased level of *Actinobacteria* was associated with SCFA production [28], while *Deferribacterota* was considered an opportunistic pathogen, which was found to increase in an experimental inflammatory bowel disease (IBD) model [29]. There are very limited reports on *Spirochaetota*, *Desulfobacterota*, and V*errucomicrobiota*, and it is uncertain whether they have adverse effects.

IR treatment significantly boosted *Alloprevotella* abundance and decreased *Allobaculum. Alloprevotella* and *Allobaculum* were both producers of SCFA, which has immunomodulatory and anti-inflammatory effects [30]. PC can significantly promote *Bacteroides*, which was reported to be a key player in the immunomodulation of the human immune system [31]. *Bacteroides* can produce eight capsular polysaccharides, the beneficial effects of capsular polysaccharides included stimulation, development, and homeostasis of the immune system [32], and prevention of bacterial and viral infections [33]. In this study, both PC and IR treatments significantly increased *Parabacteroides* abundance. Lei et al. [34] reported that *Parabacteroides* can produce acetate to alleviate heparanase-exacerbated acute pancreatitis through reducing neutrophil infiltration.

In conclusion, the microbiota composition was significantly modulated by PC and IR in healthy mice. Both PC and IR boosted the abundance of beneficial bacteria in the intestinal tract of mice, which suggested the potentially beneficial effects of PC and IR in the gut microenvironment for animals or humans in the future. Particularly, IR might inhibit the growth of short-chain fatty-acid-producing bacteria.

Short chain fatty acids (SCFA) are the principal end-products of non-digestible carbohydrate fermentation [16]. The main SCFA found in feces are acetic acid, propionic acid, and butyric acid, which have all been extensively investigated and found to be beneficial. They have been shown to affect host energy balance and have been connected to gut barrier function, immunological function, and inflammatory disease.

A study demonstrated that the activation of intestinal gluconeogenesis (IGN) by propionate and butyrate acids avoided obesity and insulin resistance in high-fat-diet-fed mice [35]. Propionate stimulated GPR41 in the periportal afferent neural pathway to result in IGN, whereas butyrate directly caused IGN in enterocytes. The increased IGN resulted in decreased hepatic glucose production and improved energy homeostasis [35]. Acetate acid appears to promote leptin secretion in adipocytes. Leptin is an important adipose-derived homeostatic signal that regulates energy balance [36]. Butyrate increases the gut barrier function by upregulating the expression of tight junction proteins and mucins, which helps to maintain gut integrity [37]. SCFA play a role in immune system and inflammatory response regulation. Propionate and butyrate have been found to play a role in regulatory T cell generation and function at the whole-animal level by inhibiting histone deacetylation [38]. A previous study has demonstrated that acetate acid mediated joint inflammation in a murine gout model through inflammasome assembly and IL-1β production that is partially FFAR2 dependent [39].

The effects of hexanoic acid or isohexanoic acid on health are poorly understood. Hexanoic acid has been proven to have cytotoxic capabilities against neoplastic cells in previous study [40]. Study on a rat model showed that in the feces of animals with nonbacterial chronic prostate inflammation (CPI), the levels of hexanoic acid were decreased, which indicated that prostate inflammation was associated with changes in hexanoic acid level [41]. Studies have confirmed that valeric acid was involved in the pathophysiology of inflammatory bowel disease (IBD) and might be a prognostic marker of the disease state. Jaworska et al. [42] found that the ratio of valeric acid in feces in patients with IBD was statistically substantially greater than in healthy people.

Carbohydrates are the primary source of acetic, propionic, and butyric acids, but the amino acids valine and leucine derived from protein degradation can be converted into isobutyric and isovaleric acids, which are referred to as branched chain SCFA [43]. Among short-chain fatty acids, branched forms account for 5–10%, making little contribution to the total SCFA yield. The effect of branched chain SCFA on health has not been sufficiently studied. A previous study showed that the isobutyric acid enhances glucose absorption and may lead to increased insulin sensitivity in persons with metabolic diseases [44]. Further research is needed in order to elucidate the mechanisms of action of branched chain SCFA in health.

Our results showed that PC supplementation significantly increased acetate acid and branched chain SCFAs levels in the feces of mice, while IR supplementation had no apparent effect on the content of SCFA, suggesting that PC may exert a health effect by regulating SCFA, and the health promoting effect of IR is independent of the SCFA pathway.

Aguirre et al. [45] reported the production of branched chain SCFA was carried out mainly by the genera *Bacteroides* and *Clostridium*. Results of this study suggested that *Alistipes*, *Anaerotrunucus*, *ASF356*, *Roseburia*, *Ruminococcaceae*, *Rikenella*, and *Parabacteroides* might also be responsible for the production of branched chain SCFA. Furthermore, supplementing with PC and IR significantly increased *Parabacteroides* abundance in the intestinal tract of mice, and also significantly increased the isobutyric acid level in the feces of mice, the correlation analysis result indicated that *Parabacteroides* was significantly positively correlated with isobutyric acid, suggesting that *Parabacteroides* may be a key isobutyric acid producer.

The metabolites affected by PC or IR treatment was successfully identified using urine metabolomics. From the results of this study, PC mainly affected the pathways that related to energy absorption and consumption, as well as amino acid (glycine, serine and threonine, etc.) metabolism, while IR mainly affected the amino acid (histidine, D-glutamine and D-glutamate, etc.) metabolism pathways. Glycine has been shown to have anti-oxidant, metabolic, and neurological protective properties [46]. Glycine was found to be negatively related to obesity and to influence lipid metabolism and cholesterol transport in a previous study [47]. The carbonyl group serine is used to synthesize purine nucleotides and deoxy thymidine phosphate. In recent years, scientists have discovered that serine is required not only for cell proliferation but also for certain functions in the central nervous system [48]. These results are vital in giving a complete understanding of the metabolic pathways in the gut that change by PC and IR intervention in diseases. However, it is necessary to confirm these affected pathways to exploit their further potential as biomarkers. Combining information with other omics, such as proteomics, may give a complete picture for future research, primarily targeting a particular pathway.

This study elucidated the effects of poncirin and isosakuranetin interventions on the gut microbiota and metabolomic profiling in mice, which provided valuable clues for future research on the health-promoting mechanisms of poncirin and isosakuranetin in citrus fruits and fermented citrus products.

## 4. Materials and Methods

### 4.1. Chemicals

Poncirin and isosakuranetin standards were purchased from Shanghai yuanye Bio-Technology Co., Ltd. (Shanghai, China). Formic acid, acetonitrile, and methanol of mass spectrometry (MS) grade were acquired from Thermo Fisher Scientific-CN (Shanghai, China). Standards of SCFAs were purchased from Sigma-Aldrich Chemical Co., Ltd. (St. Louis, MO, USA). All chemicals of analytical grade were purchased from Sigma-Aldrich Chemical Co., Ltd. (St. Louis, MO, USA).

### 4.2. Animals and Experimental Design

Thirty C57Bl/6J male mice aged 6 weeks were purchased from the SPF laboratory animal center of Huazhong Agricultural University (Wuhan, China), with the permission number of SYXK [E] 2020-0084. The animals and protocols for this study were approved (ID Number: HZAUMO-2022-0004) by The Scientific Ethics Committee of Huazhong Agricultural University. Mice were housed in a 25 ± 1 °C and humidity-controlled room with 12 h light/12 h dark cycles, with food and water ad libitum. After one week of acclimatization on a normal-chow diet, mice were randomly divided into three groups (*n* = 10): (1) In the poncirin (PC) group mice were administrated with poncirin (5 mg/kg/d) by oral gavage; (2) in the isosakuranetin (IR) group, mice were administrated with isosakuranetin (5 mg/kg/d) by oral gavage; (3) in the control group, mice were administrated with the same volume of 0.5% (*w*/*v*) sodium carboxyl methylcellulose solution. A dose of 5 mg/kg is a safe dose usually adopted to research the in vivo activity of poncirin and isosakuranetin [4,49]. Poncirin and isosakuranetin suspension were prepared by dispersion in 0.5% (*w*/*v*) sodium carboxyl methylcellulose solution. After 7 days of administration, fecal samples were collected and stored at −80 °C for later microbiota and short-chain fatty acid analysis, and then the mice were fasted in metabolic cages with free access to water for the collection of urine over 24 h.

### 4.3. Urine Metabolomics Analysis

A volume of 800 μL solution (methanol: acetonitrile = 1:1 (v:v), containing 0.02 mg/mL internal standard (L-2-chlorophenylalanine)) was added to 200 μL urine. After 30 s of vortex mixing, ultrasonic extraction was performed at low temperature for 30 min (5 °C, 40 KHz). The mixture was centrifuged at 13,000  rpm for 15 min to collect supernatant. The supernatant was transferred to a clean EP tube, and the solvent was removed by nitrogen blowing, with residue obtained. The residue was resuspended with 120 μL 50% acetonitrile. Samples were stored at −80 °C for the subsequent analysis. Measures of 20 μL supernatant from each sample were mixed together as quality control (QC) samples.

Metabolic profiling of urine was conducted on an ACQUITY UPLC HSS T3 (100 mm × 2.1 mm, 1.8 μm). The mobile phase consisted of 95% water + 5% acetonitrile (containing 0.1% formic acid) (solvent A) and 47.5% acetonitrile + 47.5% Isopropyl alcohol + 5% water (containing 0.1% formic acid) (solvent B). The gradient conditions of the mobile phase were as follows: 0–3.5 min, 0 – 24.5% B; 3.5–5.0 min, 24.5–65% B; 5.0–5.5 min, 65–100% B; 5.5–7.4 min, 100% B; 7.4–7.6 min, 100–51.5% B; 7.6–10.0 min, 51.5–0% B. The flow rate of the mobile phase was 0.6 mL/min. The injection volume was 2 μL, and the column temperature was controlled at 40 °C.

MS was conducted on a UHPLC-Q Exactive HF-X (Thermo Scientific, Waltham, MA, USA). Electron spray ionization (ESI) mass spectra of positive and negative ionization modes were obtained when the m/z scanning range was 70–1050. S-Lens voltage and extraction voltage were 50 V and ± 3.5 kV, respectively. Ion source temperature and desolvation temperatures were 425 °C and 320 °C. Cone gas flow rate was 50 L/h. High purity nitrogen was used as gas collision.

ProgenesisQI software 3.0 (WatersCorporation, Milford, CT, USA) was used to analyze the UPLC/Q-Exactive HF-X/MS data.

### 4.4. Fecal Microbiota Analysis

Fecal DNA was extracted using a DNA extraction kit (BioTeke Corporation, Beijing, China). The extracted bacterial DNA was then checked by agarose gel electrophoresis. Amplicon libraries were constructed for high-throughput sequencing using bacterial primers 341F (5′-CCTAYGGGRBGCASCAG-3′) and 806R (5′-GGACTACNNGGGTATCTAAT-3′) with a specific barcode targeting the V3–V4 hypervariable region of the bacterial 16S rRNA gene.

After PCR amplification, sequencing was performed on an Illumina Sequencer Miseq platform by Majorbio Bio-Pharm Technology Co. Ltd. (Shanghai, China). Illumina paired end reads were merged and filtered with the Quantitative Insights Into Microbial Ecology (QIIME) program (v1.9.1). All quality filtered sequencing reads were then clustered into operational taxonomic units (OTUs) with a threshold of 97% pairwise identity, using UPARSE software (v7.0.1090). For each representative sequence, the GreenGene Database (http://greengenes.secondgenome.com/ (accessed on 10 January 2022)) was used, based on the RDP Classifier (v2.11) to annotate the taxonomic information.

The OTU abundance information was normalized, utilizing a standard sequence number corresponding to the sample with the least sequences. Subsequent analyses were all performed based on these normalized output data.

### 4.5. Short-Chain Fatty Acid (SCFAs) Analysis

Fecal samples (25 mg) were suspended in 500 μL of saturated water (containing 0.5% phosphoric acid). After 3 min of vortex mixing, the mixture was centrifuged at 6054× *g* for 15 min to collect supernatant. Subsequently, 200 μL n-butyl alcohol (containing the internal standard 2-ethylbutyric acid (10 μg/mL)) was added to the supernatant. After 10 s of vortex mixing, ultrasonic extraction was performed for 10 min. Finally, the mixture was centrifuged at 6054× *g* for 5 min to collect supernatant for analysis.

Analysis was conducted by 8890B-5977B GC/MSD (Agilent Technologies Inc., Santa Clara, CA, USA), with an injection volume of 1 μL. The resulting chromatograms were processed using the Masshunter software 10.0.707.0 (Agilent Technologies Inc., Santa Clara, CA, USA).

### 4.6. Statistic Analysis

Data were expressed as means ± SD (*n* = 10). Statistical significances among treatment groups were analyzed with a one-way ANOVA followed by Duncan’s multiple range test using SPSS 22.0. A value of *p* < 0.05, or *p* < 0.01, or *p* < 0.001 was regarded as a significant difference or extremely significant difference, respectively.

## 5. Conclusions

This study revealed that poncirin and isosakuranetin had differential effects on the gut microbiota, SCFA, and urinary metabolomes in mice. Poncirin can effectively increase *Parabacteroides* and *Bacteroides* abundances in the intestines of mice, as well as the metabolites (acetic, isobutyric, and isovaleric acids) derived from gut microbiota. Isosakuranetin can significantly increase *Parabacteroides* and *Alloprevotella* and decrease *Allobaculum*, and has no significant effect on short-chain fatty acid content.

Urinary metabolomics studies elucidated the interventional effects of poncirin and isosakuranetin on metabolomic profiling. A total of 144 differential metabolites were found in the poncirin group compared with control group. These metabolites were involved in the metabolic pathways of amino acid metabolism, protein digestion and absorption, gastric acid secretion, the sphingolipid signaling pathway, insulin resistance, thermogenesis, and carbohydrate digestion and absorption. There was a total of 175 differential metabolites in the isosakuranetin group compared with the control group. These metabolites were mainly involved in the metabolic pathways of amino acid metabolism. These potential metabolites and the pathways in which they participate may become valuable clues for the focus of future research on the health promoting mechanism of poncirin and isosakuranetin.

## Data Availability

Not applicable.

## Supplementary Materials

The following supporting information can be downloaded at: https://www.mdpi.com/article/10.3390/molecules27113641/s1, Table S1: Identification of the most discriminant metabolomic variables between PC group and control group; Table S2: Identification of the most discriminant metabolomic variables between IR group and control group; Table S3: Identification of the most discriminant metabolomic variables between PC group and IR group.
