# Supplementary material for: Effects of Poncirin, a Citrus Flavonoid and Its Aglycone, Isosakuranetin, on the Gut Microbial Diversity and Metabolomics in Mice
Source: Molecules. 2022 Jun 6;27(11):3641. doi: 10.3390/molecules27113641 (PMC9182171; doi:10.3390/molecules27113641)
Supplement: Supplementary file 1 [file molecules-27-03641-s001.zip › molecules-1735342-supplementary.pdf]

Supplementary Table S1 Identification of the most discriminant metabolomic variables between PC group and Control group

m/z	Mode	Retention time	Adducts	Formula	Metabolite	Subclass	Fold change (PC/Control)	P value
407.2789	neg	6.412	M-H, M+Cl, M+FA-H, 2M-H	$C_{24}H_{40}O_5$	Cholic acid	Bile acids, alcohols and derivatives	1.177	0.001
227.0663	neg	2.628	M-H, M+FA-H, M+Cl	$C_9H_{12}N_2O_5$	2'-Deoxyuridine	Pyrimidine 2'-deoxyribonucleosides	0.899	0.041
296.6158	neg	6.172	M-2H, M-H, M+Na-2H	$C_{26}H_{45}NO_{10}S_2$	Taurocholic acid 3-sulfate	Bile acids, alcohols and derivatives	1.163	0.001
405.2631	neg	6.425	M-H, M+Cl, 2M-H	$C_{24}H_{38}O_5$	7-Ketodeoxycholic acid	Bile acids, alcohols and derivatives	1.181	0.002
461.1074	neg	6.152	M-H, 2M-H, M+Na-2H	$C_{22}H_{22}O_{11}$	6-{3,5-dihydroxy-4-[3-(4-methoxyphenyl)prop-2-enoyl]phenoxy}-3,4,5-trihydroxyoxane-2-carboxylic acid	Flavonoid glycosides	1.191	0.021
174.0395	neg	1.194	M-H2O-H, M-H	$C_6H_9NO_5$	N-acetylaspartate	Amino acids, peptides, and analogues	0.936	0.028
391.2843	neg	6.528	M-H, M+FA-H	$C_{24}H_{40}O_4$	Deoxycholic acid	Bile acids, alcohols and derivatives	1.203	0.014
327.1074	neg	6.172	M-H2O-H, M+FA-H	$C_{14}H_{18}O_6$	2-[4,6-dihydroxy-3-(4-hydroxy-3-methylbut-2-en-1-yl)-2-methoxyphenyl]acetic acid	Phenylacetic acids	0.880	0.003
269.1247	neg	2.476	M+FA-H	$C_{12}H_{18}NO_3^+$	(4-Hydroxybenzoyl)choline	Benzoic acids and derivatives	0.952	0.033

146.0234	neg	2.490	M-H_2O-H	$C_8H_7NO_3$	4-Pyridoxolactone	Pyridinecarboxylic acids and derivatives	1.074	0.042
282.0833	neg	2.717	M-H	$C_{10}H_{13}N_5O_5$	Guanosine	_	1.115	0.005
291.0171	neg	2.875	M+FA-H	$C_9H_{10}O_6S$	{[3-(2-hydroxyphenyl)oxiran-2-yl]methoxy}sulfonic acid	1-hydroxy-4-unsubstituted benzenoids	0.939	0.010
193.0607	neg	3.019	M-H	$C_9H_{10}N_2O_3$	Aminohippuric acid	Benzoic acids and derivatives	1.055	0.012
391.1597	neg	3.455	M+FA-H	$C_{16}H_{26}O_8$	(1R,3S,4S,6R)-6,9-Dihydroxyfenchone 6-O-b-D-glucoside	Terpene glycosides	1.101	0.021
467.1864	neg	4.232	M-H_2O-H	$C_{18}H_{34}N_2O_{13}$	Glucosylgalactosyl hydroxylysine	Fatty acyl glycosides	1.051	0.007
395.2059	neg	5.077	M+FA-H	$C_{20}H_{30}O_5$	Resolvin E1	Eicosanoids	1.136	0.010
271.0602	neg	5.234	M-H	$C_{15}H_{12}O_5$	(+/-)-Naringenin	-	0.827	0.019
431.1907	neg	5.275	M+FA-H	$C_{19}H_{30}O_8$	Corchoionol C 9-glucoside	Fatty acyl glycosides	1.037	0.025
379.211	neg	5.378	M+FA-H	$C_{20}H_{30}O_4$	(ent-16betaOH)-16,17-Dihydroxy-9(11)-kauren-19-oic acid	_	1.040	0.046
347.1699	neg	5.412	M-H_2O-H	$C_{16}H_{30}O_9$	(1S,2S,4R,8S)-p-Menthane-1,2,8,9-tetrol 2-glucoside	Terpene glycosides	1.059	0.035
369.1177	neg	5.419	M-H	$C_{17}H_{22}O_9$	6-[2,4-dihydroxy-3-(3-methylbut-2-en-1-yl)phenoxy]-3,4,5-trihydroxyoxane-2-carboxylic acid	Carbohydrates and carbohydrate conjugates	0.835	0.009
345.1692	neg	5.515	M-H_2O-H	$C_{20}H_{28}O_6$	Blumealactone A	Monoterpenoids	2.203	0.018
226.0267	neg	5.659	M+Cl	$C_{10}H_9NO_3$	5-Phenyl-1,3-oxazinane-2,4-dione	_	1.146	0.030
149.0594	neg	5.686	M-H	$C_9H_{10}O_2$	4-(prop-2-en-1-yl)benzene-1,2-diol	Benzenediols	0.921	0.026
405.1752	neg	5.919	2M-H	$C_7H_{13}N_3O_4$	Glycyl-Gamma-glutamate	Amino acids, peptides, and analogues	0.957	0.007
231.0322	neg	6.138	M-H	$C_9H_{12}O_5S$	(4-ethyl-2-methoxyphenyl)oxidanesulfonic acid	Arylsulfates	0.734	0.000

471.3098	neg	6.460	M+FA-H	$C_{28}H_{42}O_3$	(3beta,5alpha,6alpha,7alpha,22E,24R)-5,6-Epoxyergosta-8,14,22-triene-3,7-diol	Ergostane steroids	1.143	0.010
450.2603	neg	6.665	M-H	$C_{21}H_{42}NO_7P$	LysoPE(16:1(9Z)/0:0)	Glycerophosphoethanolamines	1.450	0.000
478.2921	neg	6.789	M-H	$C_{23}H_{46}NO_7P$	PE(18:1(9Z)/0:0)	-	1.430	0.000
509.2866	neg	7.648	M+FA-H	$C_{23}H_{45}O_7P$	1-(11Z-eicosenoyl)-glycero-3-phosphate	Glycerophosphates	1.324	0.000
436.2817	neg	6.844	M-H	$C_{21}H_{44}NO_6P$	PE(P-16:0e/0:0)	-	1.084	0.042
452.2767	neg	6.782	M-H	$C_{21}H_{44}NO_7P$	PE(16:0/0:0)	Glycerophosphoethanolamines	1.565	0.000
498.2878	neg	6.727	M+FA-H	$C_{21}H_{44}NO_7P$	LysoPE(0:0/16:0)	Glycerophosphoethanolamines	1.359	0.005
455.3152	neg	6.583	M+FA-H	$C_{28}H_{42}O_2$	Gamma-Tocotrienol	Quinone and hydroquinone lipids	1.168	0.040
473.326	neg	6.501	M-H	$C_{29}H_{46}O_5$	(3beta,17alpha,23S)-17,23-Epoxy-3,28,29-trihydroxy-27-norlanost-8-en-24-one	Diterpenoids	1.122	0.031
509.217	neg	6.446	M+Na-2H	$C_{24}H_{40}O_8S$	7-Sulfocholic acid	Bile acids, alcohols and derivatives	1.245	0.004
421.2579	neg	6.412	M+FA-H	$C_{23}H_{36}O_4$	MG(0:0/20:5(5Z,8Z,11Z,14Z,17Z)/0:0)	_	1.139	0.014
501.2848	neg	6.398	M-H2O-H	$C_{29}H_{44}O_8$	Digoxigenin monodigitoxoside	Ethers	1.181	0.003
503.3	neg	6.391	M+FA-H	$C_{28}H_{42}O_5$	Pubesenolide	Triterpenoids	1.256	0.007
441.1749	neg	6.288	M+FA-H	$C_{20}H_{28}O_8$	3,4,5-trihydroxy-6-{[(6Z)-7-hydroxy-6-(phenylmethylidene)heptan-2-yl]oxy}oxane-2-carboxylic acid	1-hydroxy-2-unsubstituted benzenoids	0.844	0.016
379.2112	neg	6.172	M+FA-H	$C_{20}H_{30}O_4$	Prostaglandin B2	Eicosanoids	1.089	0.021
359.0763	neg	6.104	M+FA-H	$C_{17}H_{14}O_6$	2-(3,5-dimethoxyphenyl)-5,7-dihydroxy-4H-chromen-4-one	O-methylated flavonoids	1.191	0.014
223.0992	neg	5.747	M+FA-H	$C_{11}H_{14}O_2$	3-hydroxy-3-methyl-4-phenylbutan-2-one	Phenylpropanes	0.966	0.015
383.1268	neg	5.563	M+K-2H	$C_{20}H_{26}O_5$	19-Noraldosterone	Hydroxysteroids	1.134	0.019
295.1179	neg	5.501	M+FA-H	$C_{14}H_{18}O_4$	Helinorbisabone	Monoterpenoids	1.083	0.049

403.1595	neg	5.193	2M-H	$C_9H_{14}O_5$	3-(1-Hydroxymethyl-1-propenyl)pentanedioic acid	Fatty acids and conjugates	0.955	0.001
265.1071	neg	5.138	M+FA-H	$C_{13}H_{16}O_3$	(1Z)-2-hydroxy-1-(4-methoxyphenyl)-4-methylpent-1-en-3-one	Anisoles	1.032	0.017
303.0531	neg	4.825	M+FA-H	$C_{11}H_{14}O_5S$	(3-methyl-2-oxo-4-phenylbutoxy)sulfonic acid	Phenylpropanes	0.878	0.003
257.1385	neg	4.641	M+FA-H	$C_{12}H_{20}O_3$	Cucurbic acid	Lineolic acids and derivatives	1.051	0.035
327.1438	neg	4.300	M-H	$C_{16}H_{24}O_7$	PGDM	-	1.167	0.001
239.055	neg	3.761	M+FA-H	$C_{10}H_{10}O_4$	3-(4-methoxyphenyl)oxirane-2-carboxylic acid	Anisoles	0.934	0.002
206.0448	neg	3.707	M-H_2O-H	$C_{10}H_{11}NO_5$	2-{[hydroxy(2-hydroxy-4-methoxyphenyl)methylidene]amino}acetic acid	Benzoic acids and derivatives	1.054	0.002
240.0503	neg	3.489	M+FA-H	$C_9H_9NO_4$	2-Hydroxy-6,7-dimethoxybenzoxazole	Benzoxazolones	1.107	0.014
317.0324	neg	3.366	M+FA-H	$C_{11}H_{12}O_6S$	[(5-hydroxy-2-methyl-2H-chromen-2-yl)methoxy]sulfonic acid	1-benzopyrans	0.911	0.026
198.0316	neg	3.305	M-H	$C_9H_{10}ClNO_2$	P-CHLOROPHENYLALANINE	_	1.051	0.001
282.9884	neg	3.067	M+Na-2H	$C_9H_{10}O_7S$	Dihydrocaffeic acid 3-sulfate	Arylsulfates	1.114	0.007
295.1385	neg	2.964	2M-H	$C_6H_{12}O_4$	Glycerol 1-propanoate	Monoradylglycerols	1.066	0.012
225.087	neg	2.882	M-H	$C_{10}H_{14}N_2O_4$	Porphobilinogen	Amines	1.028	0.001
524.1107	neg	2.628	M+Cl	$C_{24}H_{27}NO_8S$	4-Hydroxy duloxetine glucuronide	Carbohydrates and carbohydrate conjugates	0.946	0.026
151.0248	neg	2.085	M-H	$C_5H_4N_4O_2$	Xanthine	Purines and purine derivatives	1.064	0.040
413.2677	pos	6.525	M+Na, M+K, 2M+NH_4, 2M+Na,	$C_{24}H_{38}O_4$	Pregnan-20-one, 17-(acetyloxy)-3-hydroxy-6-methyl-, (3b,5b,6a)-	-	1.114	0.001

426.323	pos	6.504	M+ACN+Na, M+H M+NH4, M+Na, 2M+NH4, 2M+H, 2M+Na	$C_{24}H_{40}O_5$	3a,7a,12b-Trihydroxy-5b-cholanoic acid	-	1.216	0.000
355.2643	pos	6.441	M+H-2H2O, M+NH4, M+ACN+Na, M+H-H2O	$C_{24}H_{38}O_4$	12-Ketodeoxycholic acid	Bile acids, alcohols and derivatives	1.103	0.000
371.259	pos	6.385	2M+NH4, 2M+Na, M+NH4, M+H-2H2O	$C_{24}H_{38}O_5$	3,7-Dihydroxy-12-oxocholanoic acid	Bile acids, alcohols and derivatives	1.117	0.003
265.1191	pos	3.374	M+H, M+K	$C_{13}H_{16}N_2O_4$	AFMK	Carbonyl compounds	0.893	0.044
243.0886	pos	5.374	M+H, M+Na	$C_{12}H_{10}N_4O_2$	Lumichrome	-	1.047	0.009
197.0817	pos	5.430	M+CH3OH+H, 2M+NH4	$C_9H_8O_3$	4-methoxy-1-benzofuran-6-ol	_	0.942	0.036
336.1812	pos	6.183	M+H-H2O, M+CH3OH+H	$C_{11}H_{21}N_5O_5$	Glutamylarginine	Amino acids, peptides, and analogues	0.934	0.030
151.076	pos	6.037	M+H-H2O, M+ACN+Na	$C_9H_{12}O_3$	1-Ipomeanol	_	0.957	0.044
169.0866	pos	5.604	M+H-2H2O, M+H	$C_9H_{12}O_3$	4-Hydroxy-3-methoxyphenethyl alcohol	Methoxyphenols	0.964	0.005

185.0817	pos	3.576	M+H-H_2O, M+CH_3OH+H	$C_8H_8O_3$	Methyl furfuracrylate	Fatty acid esters	0.924	0.042
141.0107	pos	3.304	M+H, M+ACN+H	C_7H_5ClO	4-Chlorobenzaldehyde	-	1.036	0.000
263.1038	pos	3.158	M+H, M+NH_4	$C_{10}H_{18}N_2O_4S$	Methionyl-Hydroxyproline	Amino acids, peptides, and analogues	1.078	0.004
231.0984	pos	1.180	M+H, M+K	$C_9H_{14}N_2O_5$	Aspartyl-Proline	Amino acids, peptides, and analogues	0.929	0.031
154.0981	pos	1.146	M+H-H_2O, M+H	$C_7H_{11}N_3O$	Nomega-Acetylhistamine	Carboxylic acid derivatives	1.055	0.044
255.1349	pos	1.898	M+NH_4, M+ACN+H	$C_{10}H_{15}NO_4$	2-(2,6-dihydroxy-3,4-dimethoxycyclohexylidene)acetonitrile	Alcohols and polyols	0.952	0.020
240.11	pos	2.393	M+H, M+Na	$C_9H_{13}N_5O_3$	7,8-dihydro-L-Biopterin	Pterins and derivatives	0.961	0.013
400.1477	pos	2.602	M+Na, 2M+NH_4	$C_{10}H_9NO_3$	Xi-2,3-Dihydro-2-oxo-1H-indole-3-acetic acid	Indolyl carboxylic acids and derivatives	1.308	0.036
133.0978	pos	0.714	M+H	$C_5H_{12}N_2O_2$	D-Ornithine	Amino acids, peptides, and analogues	1.086	0.003
162.1131	pos	0.979	M+H	$C_7H_{15}NO_3$	L-Carnitine	Quaternary ammonium salts	1.057	0.008
132.0774	pos	1.007	M+H	$C_4H_9N_3O_2$	Creatine	Amino acids, peptides, and analogues	0.956	0.038
365.1066	pos	1.007	M+Na	$C_{12}H_{22}O_{11}$	Sucrose	Carbohydrates and carbohydrate conjugates	1.166	0.004
278.0658	pos	2.400	M+NH_4	$C_6H_{13}O_9P$	Dolichyl phosphate D-mannose	Polyprenols	0.810	0.007
298.1152	pos	2.476	M+H	$C_{11}H_{15}N_5O_5$	7-Methylguanosine	_	0.868	0.009
442.0996	pos	2.741	2M+ACN+H	$C_8H_8O_6$	2,4,5-trihydroxy-3-methoxybenzoic acid	Quinone and hydroquinone lipids	1.077	0.021
232.1191	pos	2.755	M+H	$C_{10}H_{17}NO_5$	Isovalerylglutamic acid	Amino acids, peptides, and analogues	1.106	0.007
231.1713	pos	3.019	M+H	$C_{11}H_{22}N_2O_3$	Leucyl-Valine	Amino acids, peptides, and analogues	1.045	0.048
179.071	pos	3.054	M+H	$C_{10}H_{10}O_3$	(R)-(-)-Mellein	-	0.978	0.000
313.1404	pos	3.339	M+H	$C_{19}H_{20}O_4$	M-(beta-Acetyl-alpha-ethyl-p-hydroxyphenethyl)benzoic acid	-	0.955	0.025

278.1146	pos	3.611	M+H-H_2O	$C_{13}H_{17}N_3O_5$	Asparaginyl-Tyrosine	Amino acids, peptides, and analogues	1.109	0.017
195.0773	pos	3.917	M+H	$C_9H_{10}N_2O_3$	2-Pyridylacetylglycine	-	0.942	0.030
158.0607	pos	4.439	M+H-H_2O	$C_{10}H_9NO_2$	Indoleacetic acid	Indolyl carboxylic acids and derivatives	0.948	0.003
410.1821	pos	4.683	M+ACN+H	$C_{18}H_{24}O_8$	3,4,5-trihydroxy-6-{[(1E)-1-(4-methoxyphenyl)pent-1-en-3-yl]oxy}oxane-2-carboxylic acid	Carbohydrates and carbohydrate conjugates	0.812	0.016
299.1289	pos	4.718	M+H	$C_{18}H_{18}O_4$	(+/-)-Enterolactone	Tetrahydrofuran lignans	0.932	0.006
558.2721	pos	5.018	2M+ACN+H	$C_{11}H_{18}N_2O_5$	Gamma-L-Glutamyl-L-pipecolic acid	Amino acids, peptides, and analogues	1.122	0.025
133.0654	pos	5.025	M+H	C_9H_8O	2-Indanone	-	0.968	0.001
273.0767	pos	5.213	M+H	$C_{15}H_{12}O_5$	Naringenin chalcone	Chalcones and dihydrochalcones	0.937	0.032
310.2024	pos	5.834	M+NH_4	$C_{17}H_{24}O_4$	Acetylbalchanolide	Terpene lactones	1.054	0.006
287.0926	pos	5.890	M+H	$C_{16}H_{14}O_5$	Sakuranetin	O-methylated flavonoids	1.053	0.016
210.1127	pos	6.002	M+ACN+H	$C_9H_{12}O_3$	4-Ipomeanol	Carbonyl compounds	0.951	0.004
370.1671	pos	6.093	M+NH_4	$C_{21}H_{20}O_5$	Artonol A	1-benzopyrans	1.150	0.002
300.218	pos	6.100	M+H-H_2O	$C_{16}H_{31}NO_5$	3-hydroxynonanoyl carnitine	Fatty acid esters	1.036	0.001
467.3141	pos	6.392	M+H-H_2O	$C_{30}H_{44}O_5$	Ganolucidic acid E	Triterpenoids	1.139	0.001
389.2696	pos	6.399	M+H	$C_{24}H_{36}O_4$	1alpha-hydroxy-25,26,27-trinorvitamin D3 24-carboxylic acid	-	1.159	0.008
151.1124	pos	6.406	M+H	$C_{10}H_{14}O$	Carvone	Monoterpenoids	0.931	0.000
437.2665	pos	6.659	M+H	$C_{21}H_{41}O_7P$	1-Oleoyl Lysophosphatidic Acid (sodium salt)	-	1.226	0.012
540.2988	pos	6.750	M+H-2H_2O	$C_{32}H_{41}N_5O_5$	Mauritine A	Amino acids, peptides, and analogues	1.258	0.000
666.6055	pos	7.701	M+NH_4	$C_{41}H_{76}O_5$	DG(20:1(11Z)/18:1(11Z)/0:0)	Diradylglycerols	1.031	0.000
708.5145	pos	7.526	M+CH_3OH+H	$C_{36}H_{70}NO_8P$	PE(15:0/16:1(9Z))	Glycerophosphoethanolamines	1.029	0.000

638.5743	pos	7.512	M+NH4	$C_{39}H_{72}O_5$	DG(18:0/18:2(9Z,12Z)/0:0)	Lineolic acids and derivatives	1.034	0.000
478.2947	pos	6.694	M+ACN+H	$C_{21}H_{41}O_7P$	LysoPA(0:0/18:1(9Z))	Glycerophosphates	1.123	0.017
500.2615	pos	6.638	M+ACN+H	$C_{26}H_{34}O_7$	6-hydroxy-5-{[(3-hydroxy-2-oxo-2H-chromen-7-yl)oxy]methyl}-1,1,4a,6-tetramethyl-decahydronaphthalen-2-yl acetate	Hydroxycoumarins	1.231	0.002
460.2711	pos	6.462	M+NH4	$C_{26}H_{34}O_6$	3-O-Acetylepisamarcandin	_	1.034	0.045
213.1647	pos	6.441	M+H-2H2O	$C_{16}H_{24}O_2$	2-Phenylethyl octanoate	Fatty acid esters	1.158	0.001
423.2756	pos	6.413	M+H	$C_{24}H_{38}O_6$	3alpha,7beta,12alpha-Trihydroxy-6-oxo-5alpha-cholan-24-oic Acid	-	1.159	0.021
405.2646	pos	6.406	M+H	$C_{24}H_{36}O_5$	7a,12a-Dihydroxy-3-oxo-4-cholenoic acid	Bile acids, alcohols and derivatives	1.140	0.016
218.1548	pos	6.197	M+H	$C_{14}H_{19}NO$	ETHOXYQUIN	Quinolones and derivatives	0.825	0.014
414.1569	pos	6.128	M+ACN+H	$C_{20}H_{20}O_7$	6-(2,4-dihydroxyphenyl)-2-(2,6-dihydroxyphenyl)-5-hydroxy-4-methylcyclohex-3-ene-1-carboxylic acid	Benzenediols	1.207	0.000
406.2966	pos	6.114	M+H	$C_{24}H_{39}NO_4$	N-Arachidonoyl-3-hydroxy-gamma-Aminobutyric Acid	-	1.187	0.007
299.0929	pos	5.437	M+H-H2O	$C_{17}H_{16}O_6$	5,7-dihydroxy-6-methoxy-2-(4-methoxyphenyl)-3,4-dihydro-2H-1-benzopyran-4-one	O-methylated flavonoids	1.206	0.004
134.0971	pos	5.088	M+H	$C_9H_{11}N$	2-Methylindoline	-	0.925	0.014
252.1604	pos	5.081	M+ACN+H	$C_{12}H_{18}O_3$	Jasmonic acid	Lineolic acids and derivatives	0.966	0.045
372.2418	pos	4.342	2M+3H2O+2H	$C_{15}H_{30}N_6O_4$	Kinetensin 1-3	Amino acids, peptides, and analogues	1.214	0.001
155.1073	pos	4.314	M+H	$C_9H_{14}O_2$	2,6-nonadienoic acid	-	0.959	0.009
347.1689	pos	4.084	M+ACN+H	$C_{15}H_{19}N_3O_4$	Threoninyl-Tryptophan	Amino acids, peptides, and analogues	1.096	0.014
191.1186	pos	4.070	M+H	$C_{11}H_{14}N_2O$	CYTISINE	-	0.785	0.008

192.1027	pos	4.070	M+H	$C_{11}H_{13}NO_2$	5-Methoxytryptophol	Indoles	0.830	0.008
326.1974	pos	3.903	M+NH4	$C_{17}H_{24}O_5$	Dihydrocumambrin A	Terpene lactones	1.073	0.026
269.1504	pos	3.840	M+H	$C_{13}H_{20}N_2O_4$	Dihydroxymelphalan	-	0.940	0.007
208.0613	pos	3.701	M+H	$C_{10}H_9NO_4$	2-Formaminobenzoylacetate	-	1.038	0.003
182.0456	pos	3.666	M+H	$C_8H_7NO_4$	2-Methyl-3-hydroxy-5-formylpyridine-4-carboxylate	Pyridinecarboxylic acids and derivatives	0.915	0.045
127.0397	pos	3.430	M+H	$C_6H_6O_3$	4-HYDROXY-6-METHYLPYRAN-2-ONE	-	1.066	0.003
294.1454	pos	3.284	M+ACN+H	$C_{12}H_{16}N_2O_4$	3'-Hydroxyhexobarbital	Pyrimidines and pyrimidine derivatives	0.938	0.013
122.0278	pos	3.200	M+H	$C_3H_7NO_2S$	L-Cysteine	Amino acids, peptides, and analogues	0.929	0.008
276.0699	pos	3.082	M+H	$C_{10}H_{13}NO_8$	3-beta-D-Glucopyranuronosyloxy-5-methylisoxazole	-	1.316	0.004
203.1399	pos	2.887	M+H	$C_9H_{18}N_2O_3$	ALANYL-dl-LEUCINE	-	1.089	0.007
456.2106	pos	2.790	M+H-H_2O	$C_{25}H_{27}N_7O_3$	CD 1790	Purines and purine derivatives	1.077	0.013
256.0822	pos	2.783	M+H	$C_{11}H_{13}NO_6$	Nicotinate D-ribonucleoside	-	0.960	0.036
196.0838	pos	2.393	M+H	$C_7H_9N_5O_2$	2-Amino-4-hydroxy-6-(hydroxymethyl)-7,8-dihydropteridine	-	0.946	0.008
248.1502	pos	2.197	M+H	$C_{11}H_{21}NO_5$	(R)-3-hydroxybutyrylcarnitine	Fatty acid esters	1.067	0.008
200.1039	pos	2.190	M+H	$C_8H_{13}N_3O_3$	Gamma-Glutamyl-beta-aminopropiononitrile	Amino acids, peptides, and analogues	0.963	0.024
204.1239	pos	1.173	M+H	$C_9H_{17}NO_4$	Acetylcarnitine	Fatty acid esters	1.131	0.000
207.1711	pos	1.035	M+CH_3OH+H	$C_8H_{18}N_2O_2$	Ne,Ne dimethyllysine	Amino acids, peptides, and analogues	0.878	0.001
118.0659	pos	3.298	M+H	C_8H_7N	Indole	Indoles	1.024	0.000

Supplementary Table S2 Identification of the most discriminant metabolomic variables between IR group and Control group

**m/z**	**Mode**	**Retention time**	**Adducts**	**Formula**	**Metabolite**	**Subclass**	**Fold change (PC/Control)**	**P value**
407.2789	neg	6.412	M-H, M+Cl, M+FA-H, 2M-H	$C_{24}H_{40}O_5$	Cholic acid	Bile acids, alcohols and derivatives	1.135	0.005
283.0813	neg	4.539	M-H, 2M-H, M-H_2O-H	$C_{13}H_{16}O_7$	P-Cresol glucuronide	Carbohydrates and carbohydrate conjugates	1.061	0.032
296.6158	neg	6.172	M-2H, M-H, M+Na-2H	$C_{26}H_{45}NO_{10}S_2$	Taurocholic acid 3-sulfate	Bile acids, alcohols and derivatives	1.079	0.003
405.2631	neg	6.425	M-H, M+Cl, 2M-H	$C_{24}H_{38}O_5$	7-Ketodeoxycholic acid	Bile acids, alcohols and derivatives	1.147	0.008
311.2218	neg	6.631	M-H, M+Na-2H, M-H_2O-H	$C_{18}H_{32}O_4$	9,10-DiHODE	Lineolic acids and derivatives	1.099	0.049
461.1074	neg	6.152	M-H, 2M-H, M+Na-2H	$C_{22}H_{22}O_{11}$	6-{3,5-dihydroxy-4-[3-(4-methoxyphenyl)prop-2-enoyl]phenoxy}-3,4,5-trihydroxyoxane-2-carboxylic acid	Flavonoid glycosides	1.484	0.000

289.0378	neg	4.198	M-H, M+Na-2H	$C_{11}H_{14}O_7S$	(5,7-dihydroxy-2,2-dimethyl-3,4-dihydro-2H-1-benzopyran-4-yl)oxidanesulfonic acid	1-benzopyrans	0.958	0.045
391.2843	neg	6.528	M-H, M+FA-H	$C_{24}H_{40}O_4$	Deoxycholic acid	Bile acids, alcohols and derivatives	1.187	0.009
307.1904	neg	6.583	M-H2O-H, M-H	$C_{18}H_{28}O_4$	Corchorifatty acid A	Lineolic acids and derivatives	1.092	0.026
327.1074	neg	6.172	M-H2O-H, M+FA-H	$C_{14}H_{18}O_6$	2-[4,6-dihydroxy-3-(4-hydroxy-3-methylbut-2-en-1-yl)-2-methoxyphenyl]acetic acid	Phenylacetic acids	0.858	0.001
216.0502	neg	1.187	M-H	$C_8H_{11}NO_6$	Lycoperdic acid	Amino acids, peptides, and analogues	0.932	0.028
362.0495	neg	2.449	M-H	$C_{10}H_{14}N_5O_8P$	Guanidylic acid (guanosine monophosphate)	-	1.203	0.021
269.1247	neg	2.476	M+FA-H	$C_{12}H_{18}NO_3+$	(4-Hydroxybenzoyl)choline	Benzoic acids and derivatives	0.943	0.020
146.0234	neg	2.490	M-H2O-H	$C_8H_7NO_3$	4-Pyridoxolactone	Pyridinecarboxylic acids and derivatives	1.066	0.025
282.0833	neg	2.717	M-H	$C_{10}H_{13}N_5O_5$	Guanosine	_	1.097	0.012
188.9851	neg	3.107	M-H	$C_6H_6O_5S$	Pyrocatechol sulfate	Arylsulfates	0.965	0.028
269.0657	neg	3.563	M-H	$C_{12}H_{14}O_7$	Phenyl glucuronide	Carbohydrates and carbohydrate conjugates	1.114	0.027
153.018	neg	3.734	M-H	$C_7H_6O_4$	Gentisic acid	Benzoic acids and derivatives	0.899	0.042
327.1074	neg	4.055	M-H2O-H	$C_{15}H_{22}O_9$	Aucubin	Terpene glycosides	0.842	0.029
593.1492	neg	4.109	M-H2O-H	$C_{27}H_{32}O_{16}$	6-{[3,5-dihydroxy-2-(3-hydroxyphenyl)-6-[3,4,5-trihydroxy-6-(hydroxymethyl)oxan-2-yl]-3,4-dihydro-	Flavonoid glycosides	1.213	0.005

					2H-1-benzopyran-7-yl]oxy}-3,4,5-trihydroxyoxane-2-carboxylic acid			
387.1646	neg	4.218	M+FA-H	$C_{17}H_{26}O_7$	Jasmolone glucoside	Carbohydrates and carbohydrate conjugates	0.871	0.043
497.0742	neg	4.389	M-H	$C_{21}H_{22}O_{12}S$	3-{7-[(6-carboxy-3,4,5-trihydroxyoxan-2-yl)oxy]-3-hydroxy-5-sulfino-3,4-dihydro-2H-1-benzopyran-2-yl}benzen-1-olate	Flavonoid glycosides	1.087	0.012
417.1399	neg	4.505	M+Na-2H	$C_{22}H_{24}N_2O_5$	Benazeprilat	Amino acids, peptides, and analogues	0.887	0.041
345.063	neg	4.798	M-H	$C_{14}H_{18}O_8S$	3-hydroxy-3-[4-hydroxy-3-(3-methylbut-2-en-1-yl)phenyl]-2-(sulfooxy)propanoic acid	_	1.244	0.000
271.0602	neg	5.234	M-H	$C_{15}H_{12}O_5$	(+/-)-Naringenin	-	0.831	0.029
431.1907	neg	5.275	M+FA-H	$C_{19}H_{30}O_8$	Corchoionol C 9-glucoside	Fatty acyl glycosides	1.053	0.002
347.1699	neg	5.412	M-H2O-H	$C_{16}H_{30}O_9$	(1S,2S,4R,8S)-p-Menthane-1,2,8,9-tetrol 2-glucoside	Terpene glycosides	1.069	0.008
369.1177	neg	5.419	M-H	$C_{17}H_{22}O_9$	6-[2,4-dihydroxy-3-(3-methylbut-2-en-1-yl)phenoxy]-3,4,5-trihydroxyoxane-2-carboxylic acid	Carbohydrates and carbohydrate conjugates	0.739	0.001
306.1184	neg	5.645	M+FA-H	$C_{11}H_{19}NO_6$	Epidermin	Carbohydrates and carbohydrate conjugates	1.279	0.049
405.1752	neg	5.919	2M-H	$C_7H_{13}N_3O_4$	Glycyl-Gamma-glutamate	Amino acids, peptides, and analogues	0.949	0.002
231.0322	neg	6.138	M-H	$C_9H_{12}O_5S$	(4-ethyl-2-methoxyphenyl)oxidanesulfonic acid	Arylsulfates	0.868	0.000

471.3098	neg	6.460	M+FA-H	$C_{28}H_{42}O_3$	(3beta,5alpha,6alpha,7alpha,22E,24R)-5,6-Epoxyergosta-8,14,22-triene-3,7-diol	Ergostane steroids	1.148	0.004
645.4927	neg	6.480	2M+FA-H	$C_{21}H_{32}O$	Cardanoldiene	1-hydroxy-4-unsubstituted benzenoids	1.102	0.034
271.1906	neg	6.542	M+FA-H	$C_{14}H_{26}O_2$	Myristoleic acid	Fatty acids and conjugates	1.122	0.026
485.3256	neg	6.590	M-H	$C_{30}H_{46}O_5$	Lucyin A	Triterpenoids	1.157	0.015
323.2216	neg	6.624	M-H	$C_{19}H_{32}O_4$	TOFA	-	1.157	0.033
453.2993	neg	6.638	M+Na-2H	$C_{27}H_{44}O_4$	(25R)-3beta,4beta-dihydroxycholest-5-en-26-oate(1-)	Bile acids, alcohols and derivatives	1.168	0.013
450.2603	neg	6.665	M-H	$C_{21}H_{42}NO_7P$	LysoPE(16:1(9Z)/0:0)	Glycerophosphoethanolamines	1.220	0.024
478.2921	neg	6.789	M-H	$C_{23}H_{46}NO_7P$	PE(18:1(9Z)/0:0)	-	1.186	0.038
462.2974	neg	6.858	M-H_2O-H	$C_{23}H_{48}NO_7P$	LysoPE(18:0/0:0)	Glycerophosphoethanolamines	1.149	0.007
271.227	neg	6.878	M-H	$C_{16}H_{32}O_3$	2-hydroxyhexadecanoic acid	Fatty acids and conjugates	1.077	0.031
299.2581	neg	7.002	M-H	$C_{18}H_{36}O_3$	DL-2-hydroxy stearic acid	-	1.104	0.010
436.2817	neg	6.844	M-H	$C_{21}H_{44}NO_6P$	PE(P-16:0e/0:0)	-	1.154	0.000
452.2767	neg	6.782	M-H	$C_{21}H_{44}NO_7P$	PE(16:0/0:0)	Glycerophosphoethanolamines	1.301	0.021
498.2878	neg	6.727	M+FA-H	$C_{21}H_{44}NO_7P$	LysoPE(0:0/16:0)	Glycerophosphoethanolamines	1.314	0.011
455.3152	neg	6.583	M+FA-H	$C_{28}H_{42}O_2$	Gamma-Tocotrienol	Quinone and hydroquinone lipids	1.268	0.000
473.326	neg	6.501	M-H	$C_{29}H_{46}O_5$	(3beta,17alpha,23S)-17,23-Epoxy-3,28,29-trihydroxy-27-norlanost-8-en-24-one	Diterpenoids	1.156	0.001
435.2736	neg	6.480	M+FA-H	$C_{24}H_{38}O_4$	12alpha-hydroxy-3-oxo-5beta-cholan-24-oic Acid	Bile acids, alcohols and derivatives	1.109	0.029
509.217	neg	6.446	M+Na-2H	$C_{24}H_{40}O_8S$	7-Sulfocholic acid	Bile acids, alcohols and derivatives	1.195	0.019

421.2579	neg	6.412	M+FA-H	$C_{23}H_{36}O_4$	MG(0:0/20:5(5Z,8Z,11Z,14Z,17Z)/0:0)	_	1.119	0.025
283.0603	neg	6.412	M-H	$C_{16}H_{12}O_5$	Acacetin	O-methylated flavonoids	1.286	0.001
501.2848	neg	6.398	M-H_2O-H	$C_{29}H_{44}O_8$	Digoxigenin monodigitoxoside	Ethers	1.124	0.011
503.3	neg	6.391	M+FA-H	$C_{28}H_{42}O_5$	Pubesenolide	Triterpenoids	1.211	0.013
517.3152	neg	6.371	M+FA-H	$C_{29}H_{44}O_5$	(3beta,17alpha,23R)-17,23-Epoxy-3,29-dihydroxy-27-norlanost-8-ene-15,24-dione	Triterpenoids	1.168	0.037
441.1749	neg	6.288	M+FA-H	$C_{20}H_{28}O_8$	3,4,5-trihydroxy-6-{[(6Z)-7-hydroxy-6-(phenylmethylidene)heptan-2-yl]oxy}oxane-2-carboxylic acid	1-hydroxy-2-unsubstituted benzenoids	0.822	0.002
309.1333	neg	6.240	M+FA-H	$C_{15}H_{20}O_4$	Tavulin	Terpene lactones	0.837	0.010
671.3966	neg	6.165	M+Cl	$C_{36}H_{60}O_9$	Ginsenoside Rh7	Triterpenoids	1.164	0.009
397.1125	neg	6.145	M-H_2O-H	$C_{18}H_{24}O_{11}$	3,4,5-trihydroxy-6-{[4-hydroxy-5-(4-hydroxy-3-methoxyphenyl)pentanoyl]oxy}oxane-2-carboxylic acid	Carbohydrates and carbohydrate conjugates	0.890	0.042
239.0915	neg	6.056	M-H	$C_{12}H_{16}O_5$	CMPF	Fatty acids and conjugates	0.941	0.009
263.1279	neg	5.816	M-H	$C_{15}H_{20}O_4$	(+/-)Abscisic Acid	-	0.897	0.041
399.128	neg	5.768	M+FA-H	$C_{17}H_{22}O_8$	Methyl helianthenoate F glucoside	Fatty acyl glycosides	0.834	0.035
359.1699	neg	5.631	M+FA-H	$C_{16}H_{26}O_6$	Perilloside A	Terpene glycosides	1.061	0.023
248.0918	neg	5.138	M+FA-H	$C_{12}H_{13}NO_2$	5-(2-Furanyl)-1,2,3,4,5,6-hexahydro-7H-cyclopenta[b]pyridin-7-one	Hydropyridines	0.937	0.039
376.1424	neg	4.976	M+FA-H	$C_{18}H_{21}NO_5$	2,5-dihydroxy-4-(2-hydroxyphenyl)-5-(methylamino)-3-phenylpentanoic acid	_	1.077	0.003
195.0651	neg	4.600	M-H	$C_{10}H_{12}O_4$	Homoveratric acid	Methoxybenzenes	1.058	0.042

351.0683	neg	4.546	M+Na-2H	$C_{14}H_{18}O_9$	6-(5-ethyl-2,3-dihydroxyphenoxy)-3,4,5-trihydroxyoxane-2-carboxylic acid	Carbohydrates and carbohydrate conjugates	1.110	0.044
363.1649	neg	4.170	M-H_2O-H	$C_{16}H_{30}O_{10}$	1,2,10-Trihydroxydihydro-trans-linalyl oxide 7-O-beta-D-glucopyranoside	Carbohydrates and carbohydrate conjugates	1.032	0.030
463.0865	neg	4.048	M-H	$C_{21}H_{20}O_{12}$	3,4,5-trihydroxy-6-{4-[3-oxo-3-(2,3,4,6-tetrahydroxyphenyl)prop-1-en-1-yl]phenoxy}oxane-2-carboxylic acid	Flavonoid glycosides	0.804	0.009
239.055	neg	3.761	M+FA-H	$C_{10}H_{10}O_4$	3-(4-methoxyphenyl)oxirane-2-carboxylic acid	Anisoles	0.930	0.001
206.0448	neg	3.707	M-H_2O-H	$C_{10}H_{11}NO_5$	2-{[hydroxy(2-hydroxy-4-methoxyphenyl)methylidene]amino}acetic acid	Benzoic acids and derivatives	1.032	0.015
255.0502	neg	2.984	M-H	$C_{11}H_{12}O_7$	PISCIDIC ACID	_	0.892	0.002
295.1385	neg	2.964	2M-H	$C_6H_{12}O_4$	Glycerol 1-propanoate	Monoradylglycerols	1.065	0.016
347.1447	neg	2.848	M+Cl	$C_{20}H_{24}O_3$	2-hydroxyethinylestradiol	Estrane steroids	0.962	0.020
267.0726	neg	2.717	M-H	$C_{10}H_{12}N_4O_5$	Inosine	_	1.099	0.008
524.1107	neg	2.628	M+Cl	$C_{24}H_{27}NO_8S$	4-Hydroxy duloxetine glucuronide	Carbohydrates and carbohydrate conjugates	0.951	0.031
151.0248	neg	2.085	M-H	$C_5H_4N_4O_2$	Xanthine	Purines and purine derivatives	1.081	0.009
235.0386	neg	1.166	M-H_2O-H	$C_7H_{14}N_2O_6S$	5-L-Glutamyl-taurine	Amino acids, peptides, and analogues	1.090	0.036
226.9953	neg	1.022	M+Cl	$C_6H_8O_7$	D-Glucaro-1,4-lactone	Gamma butyrolactones	0.701	0.016
413.2677	pos	6.525	M+Na, M+K, 2M+NH_4, 2M+Na,	$C_{24}H_{38}O_4$	Pregnan-20-one, 17-(acetyloxy)-3-hydroxy-6-methyl-, (3b,5b,6a)-	-	1.095	0.002

			M+ACN+Na, M+H					
174.1132	pos	5.011	M+H, 2M+H, M+2Na-H, M+Na, M+H-H_2O	$C_8H_{15}NO_3$	Hexanoylglycine	Amino acids, peptides, and analogues	1.032	0.039
426.323	pos	6.504	M+NH_4, M+Na, 2M+NH_4, 2M+H, 2M+Na	$C_{24}H_{40}O_5$	3a,7a,12b-Trihydroxy-5b-cholanoic acid	-	1.132	0.008
463.1253	pos	6.148	M+H, M+Na, M+NH_4, M+H-H_2O	$C_{22}H_{22}O_{11}$	3,4,5-trihydroxy-6-[4-(7-hydroxy-8-methoxy-4-oxo-3,4-dihydro-2H-1-benzopyran-2-yl)phenoxy]oxane-2-carboxylic acid	Flavonoid glycosides	1.450	0.000
355.2643	pos	6.441	M+H-2H_2O, M+NH_4, M+ACN+Na, M+H-H_2O	$C_{24}H_{38}O_4$	12-Ketodeoxycholic acid	Bile acids, alcohols and derivatives	1.074	0.018
371.259	pos	6.385	2M+NH_4, 2M+Na, M+NH_4, M+H-2H_2O	$C_{24}H_{38}O_5$	3,7-Dihydroxy-12-oxocholanoic acid	Bile acids, alcohols and derivatives	1.099	0.009
152.0574	pos	1.173	M+H, M+Na, M+K	$C_5H_5N_5O$	Guanine	Purines and purine derivatives	1.066	0.036

146.093	pos	1.160	M+H-H_2O, M+K, M+H	$C_5H_{11}N_3O_2$	4-Guanidinobutanoic acid	Amino acids, peptides, and analogues	0.968	0.025
303.1198	pos	2.831	M+H, M+Na	$C_{12}H_{18}N_2O_7$	Bicozamycin	-	1.079	0.034
288.1927	pos	3.270	M+H-2H_2O, M+H	$C_{18}H_{29}NO_4$	N-Jasmonoylisoleucine	Amino acids, peptides, and analogues	1.110	0.021
265.1191	pos	3.374	M+H, M+K	$C_{13}H_{16}N_2O_4$	AFMK	Carbonyl compounds	0.869	0.016
377.147	pos	3.903	M+H, M+Na	$C_{17}H_{20}N_4O_6$	Riboflavin (Vitamin B2)	-	0.950	0.001
299.0928	pos	4.858	M+H-H_2O, M+ACN+H	$C_{17}H_{16}O_6$	6-hydroxy-2-(4-hydroxyphenyl)-7,8-dimethoxy-3,4-dihydro-2H-1-benzopyran-4-one	O-methylated flavonoids	1.195	0.000
243.0886	pos	5.374	M+H, M+Na	$C_{12}H_{10}N_4O_2$	Lumichrome	-	1.055	0.001
197.0817	pos	5.430	M+CH_3OH+H, 2M+NH_4	$C_9H_8O_3$	4-methoxy-1-benzofuran-6-ol	_	0.942	0.033
482.3622	pos	7.037	M+H, M+Na	$C_{24}H_{52}NO_6P$	PC(O-16:0/0:0)	-	1.062	0.010
336.1812	pos	6.183	M+H-H_2O, M+CH_3OH+H	$C_{11}H_{21}N_5O_5$	Glutamylarginine	Amino acids, peptides, and analogues	0.917	0.009
151.076	pos	6.037	M+H-H_2O, M+ACN+Na	$C_9H_{12}O_3$	1-Ipomeanol	_	0.943	0.013
197.1293	pos	5.095	M+H-2H_2O, M+NH_4	$C_{10}H_{20}N_2O_4$	Threoninyl-Leucine	Amino acids, peptides, and analogues	0.936	0.034
185.0817	pos	3.576	M+H-H_2O, M+CH_3OH+H	$C_8H_8O_3$	Methyl furfuracrylate	Fatty acid esters	0.910	0.015
251.1399	pos	3.555	M+H, M+NH_4	$C_{13}H_{15}NO_3$	3-(4-hydroxyphenyl)-N-(4-oxobutyl)prop-2-enimidic acid	Hydroxycinnamic acids and derivatives	0.896	0.006
165.0554	pos	3.082	M+H-H_2O, M+H	$C_9H_{10}O_4$	3-Hydroxy-4-methoxyphenylacetic acid	Methoxyphenols	0.945	0.008

136.0624	pos	1.166	M+H, 2M+NH4	$C_5H_5N_5$	Adenine	Purines and purine derivatives	1.071	0.022
137.0464	pos	1.730	M+H, 2M+H	$C_5H_4N_4O$	Hypoxanthine	Purines and purine derivatives	1.085	0.018
255.1349	pos	1.898	M+NH4, M+ACN+H	$C_{10}H_{15}NO_4$	2-(2,6-dihydroxy-3,4-dimethoxycyclohexylidene)acetonitrile	Alcohols and polyols	0.944	0.008
240.11	pos	2.393	M+H, M+Na	$C_9H_{13}N_5O_3$	7,8-dihydro-L-Biopterin	Pterins and derivatives	0.944	0.004
317.0881	pos	3.026	M+H-2H2O, M+H	$C_{13}H_{16}O_9$	3,4,5-trihydroxy-6-(2-hydroxy-6-methoxyphenoxy)oxane-2-carboxylic acid	Carbohydrates and carbohydrate conjugates	1.375	0.000
133.0978	pos	0.714	M+H	$C_5H_{12}N_2O_2$	D-Ornithine	Amino acids, peptides, and analogues	1.062	0.009
144.0484	pos	0.993	M+H	C_6H_9NOS	4-Methyl-5-thiazoleethanol	Thiazoles	0.952	0.016
167.0935	pos	1.139	M+H	$C_7H_{10}N_4O$	N-Formyl-4-amino-5-aminomethyl-2-methylpyrimidine	-	0.939	0.023
278.0658	pos	2.400	M+NH4	$C_6H_{13}O_9P$	Dolichyl phosphate D-mannose	Polyprenols	0.800	0.003
298.1152	pos	2.476	M+H	$C_{11}H_{15}N_5O_5$	7-Methylguanosine	_	0.843	0.001
442.0996	pos	2.741	2M+ACN+H	$C_8H_8O_6$	2,4,5-trihydroxy-3-methoxybenzoic acid	Quinone and hydroquinone lipids	1.076	0.011
232.1191	pos	2.755	M+H	$C_{10}H_{17}NO_5$	Isovalerylglutamic acid	Amino acids, peptides, and analogues	1.085	0.002
335.1117	pos	2.963	M+H-2H2O	$C_{17}H_{22}O_9$	4-Hydroxy-5-(phenyl)-valeric acid-O-glucuronide	Carbohydrates and carbohydrate conjugates	1.062	0.022
195.1501	pos	3.005	M+H-2H2O	$C_{11}H_{22}N_2O_3$	Isoleucyl-Valine	Amino acids, peptides, and analogues	1.157	0.044
472.1594	pos	3.040	M+H	$C_{20}H_{21}N_7O_7$	10-Formyldihydrofolate	Phosphosphingolipids	0.946	0.040
179.0493	pos	3.151	M+H	$C_5H_{10}N_2O_3S$	L-Cys-Gly	Amino acids, peptides, and analogues	0.921	0.030

313.1404	pos	3.339	M+H	$C_{19}H_{20}O_4$	M-(beta-Acetyl-alpha-ethyl-p-hydroxyphenethyl)benzoic acid	-	0.949	0.007
278.1146	pos	3.611	M+H-H_2O	$C_{13}H_{17}N_3O_5$	Asparaginyl-Tyrosine	Amino acids, peptides, and analogues	1.063	0.011
195.0773	pos	3.917	M+H	$C_9H_{10}N_2O_3$	2-Pyridylacetylglycine	-	0.934	0.003
410.1821	pos	4.683	M+ACN+H	$C_{18}H_{24}O_8$	3,4,5-trihydroxy-6-{[(1E)-1-(4-methoxyphenyl)pent-1-en-3-yl]oxy}oxane-2-carboxylic acid	Carbohydrates and carbohydrate conjugates	0.760	0.023
319.1664	pos	4.899	M+K	$C_{17}H_{28}O_3$	12S-HHT	Fatty acids and conjugates	1.104	0.011
558.2721	pos	5.018	2M+ACN+H	$C_{11}H_{18}N_2O_5$	Gamma-L-Glutamyl-L-pipecolic acid	Amino acids, peptides, and analogues	1.113	0.026
170.0608	pos	5.164	M+H-2H_2O	$C_{11}H_{11}NO_3$	Edulitine	Quinolones and derivatives	0.963	0.037
319.1277	pos	5.290	M+NH_4	$C_{11}H_{15}N_3O_7$	Gamma-Glutamyl-beta-(isoxazolin-5-on-2-yl)alanine	Amino acids, peptides, and analogues	0.921	0.044
176.1078	pos	5.534	M+H	$C_{11}H_{13}NO$	N-Acetyltranylcypromine	-	0.864	0.020
287.0926	pos	5.890	M+H	$C_{16}H_{14}O_5$	Sakuranetin	O-methylated flavonoids	1.057	0.005
210.1127	pos	6.002	M+ACN+H	$C_9H_{12}O_3$	4-Ipomeanol	Carbonyl compounds	0.948	0.002
467.3141	pos	6.392	M+H-H_2O	$C_{30}H_{44}O_5$	Ganolucidic acid E	Triterpenoids	1.105	0.027
389.2696	pos	6.399	M+H	$C_{24}H_{36}O_4$	1alpha-hydroxy-25,26,27-trinorvitamin D3 24-carboxylic acid	-	1.127	0.029
151.1124	pos	6.406	M+H	$C_{10}H_{14}O$	Carvone	Monoterpenoids	0.911	0.000
285.0768	pos	6.406	M+H	$C_{16}H_{12}O_5$	Genkwanin	-	1.274	0.000
437.2665	pos	6.659	M+H	$C_{21}H_{41}O_7P$	1-Oleoyl Lysophosphatidic Acid (sodium salt)	-	1.145	0.043
540.2988	pos	6.750	M+H-2H_2O	$C_{32}H_{41}N_5O_5$	Mauritine A	Amino acids, peptides, and analogues	1.231	0.001

500.2615	pos	6.638	M+ACN+H	$C_{26}H_{34}O_7$	6-hydroxy-5-{[(3-hydroxy-2-oxo-2H-chromen-7-yl)oxy]methyl}-1,1,4a,6-tetramethyl-decahydronaphthalen-2-yl acetate	Hydroxycoumarins	1.142	0.023
330.3378	pos	6.455	M+H	$C_{20}H_{43}NO_2$	N,N-dimethyl-Safingol	-	1.038	0.036
213.1647	pos	6.441	M+H-2H2O	$C_{16}H_{24}O_2$	2-Phenylethyl octanoate	Fatty acid esters	1.143	0.003
423.2756	pos	6.413	M+H	$C_{24}H_{38}O_6$	3alpha,7beta,12alpha-Trihydroxy-6-oxo-5alpha-cholan-24-oic Acid	-	1.134	0.039
405.2646	pos	6.406	M+H	$C_{24}H_{36}O_5$	7a,12a-Dihydroxy-3-oxo-4-cholenoic acid	Bile acids, alcohols and derivatives	1.124	0.023
302.3064	pos	6.392	M+H	$C_{18}H_{39}NO_2$	Sphinganine	Amines	1.057	0.002
218.1548	pos	6.197	M+H	$C_{14}H_{19}NO$	ETHOXYQUIN	Quinolones and derivatives	0.836	0.012
385.1851	pos	5.981	M+H-H2O	$C_{19}H_{30}O_9$	D-Linalool 3-(6''-malonylglucoside)	Fatty acyl glycosides	1.051	0.014
299.0929	pos	5.437	M+H-H2O	$C_{17}H_{16}O_6$	5,7-dihydroxy-6-methoxy-2-(4-methoxyphenyl)-3,4-dihydro-2H-1-benzopyran-4-one	O-methylated flavonoids	1.100	0.042
371.169	pos	5.297	M+Na	$C_{16}H_{28}O_8$	Nepetariaside	Terpene glycosides	1.042	0.028
172.0765	pos	5.109	M+H-H2O	$C_{11}H_{11}NO_2$	Indole-3-propionic acid	Indolyl carboxylic acids and derivatives	0.966	0.035
134.0971	pos	5.088	M+H	$C_9H_{11}N$	2-Methylindoline	-	0.937	0.025
320.15	pos	5.032	M+ACN+H	$C_{15}H_{18}O_5$	2beta,9xi-Dihydroxy-8-oxo-1(10),4,11(13)-germacratrien-12,6alpha-olide	Terpene lactones	0.903	0.011
356.1169	pos	4.718	M+ACN+Na	$C_9H_{16}N_4O_7$	Canavaninosuccinate	Amino acids, peptides, and analogues	0.870	0.025

372.2418	pos	4.342	$2M+3H_2O+2H$	$C_{15}H_{30}N_6O_4$	Kinetensin 1-3	Amino acids, peptides, and analogues	1.398	0.000
347.1689	pos	4.084	M+ACN+H	$C_{15}H_{19}N_3O_4$	Threoninyl-Tryptophan	Amino acids, peptides, and analogues	1.099	0.008
191.1186	pos	4.070	M+H	$C_{11}H_{14}N_2O$	CYTISINE	-	0.829	0.016
192.1027	pos	4.070	M+H	$C_{11}H_{13}NO_2$	5-Methoxytryptophol	Indoles	0.835	0.005
197.1181	pos	4.049	M+H	$C_{11}H_{16}O_3$	Benzenemethanol, 2-(2-hydroxypropoxy)-3-methyl-	-	0.954	0.022
326.1974	pos	3.903	$M+NH_4$	$C_{17}H_{24}O_5$	Dihydrocumambrin A	Terpene lactones	1.069	0.021
269.1504	pos	3.840	M+H	$C_{13}H_{20}N_2O_4$	Dihydroxymelphalan	-	0.942	0.008
208.0613	pos	3.701	M+H	$C_{10}H_9NO_4$	2-Formaminobenzoylacetate	-	1.029	0.004
182.0456	pos	3.666	M+H	$C_8H_7NO_4$	2-Methyl-3-hydroxy-5-formylpyridine-4-carboxylate	Pyridinecarboxylic acids and derivatives	0.887	0.007
152.0713	pos	3.472	M+H	$C_8H_9NO_2$	P-Acetamidophenol (Acetaminophen, Tylenol)	-	0.943	0.039
305.0778	pos	3.444	M+ACN+H	$C_9H_{13}NO_6S$	N-acetyl-S-(3-oxo-3-carboxy-n-propyl)cysteine	Amino acids, peptides, and analogues	0.877	0.022
127.0397	pos	3.430	M+H	$C_6H_6O_3$	4-HYDROXY-6-METHYLPYRAN-2-ONE	-	1.032	0.021
374.1459	pos	3.409	$M+NH_4$	$C_{16}H_{20}O_9$	3,4,5-trihydroxy-6-{[3-(3-methoxyphenyl)propanoyl]oxy}oxane-2-carboxylic acid	Carbohydrates and carbohydrate conjugates	0.903	0.034
192.0698	pos	3.409	M+H	$C_7H_{13}NO_3S$	N-Acetyl-DL-methionine	-	0.963	0.015
315.156	pos	3.270	M+H	$C_{19}H_{22}O_4$	Grandiflorone	-	1.074	0.027
122.0278	pos	3.200	M+H	$C_3H_7NO_2S$	L-Cysteine	Amino acids, peptides, and analogues	0.928	0.010

145.1342	pos	3.054	M+H	$C_7H_{16}N_2O$	N-Acetylcadaverine	Carboxylic acid derivatives	0.931	0.010
297.1457	pos	2.866	M+H-2H$_2$O	$C_{19}H_{24}O_5$	3-[6,8-dihydroxy-2-methyl-2-(4-methylpent-3-en-1-yl)-2H-chromen-5-yl]propanoic acid	1-benzopyrans	0.956	0.040
256.0822	pos	2.783	M+H	$C_{11}H_{13}NO_6$	Nicotinate D-ribonucleoside	-	0.956	0.008
238.0944	pos	2.490	M+H	$C_9H_{11}N_5O_3$	Biopterin	Pterins and derivatives	1.029	0.008
196.0838	pos	2.393	M+H	$C_7H_9N_5O_2$	2-Amino-4-hydroxy-6-(hydroxymethyl)-7,8-dihydropteridine	-	0.924	0.004
200.1039	pos	2.190	M+H	$C_8H_{13}N_3O_3$	Gamma-Glutamyl-beta-aminopropiononitrile	Amino acids, peptides, and analogues	0.967	0.031
278.0659	pos	2.114	M+K	$C_9H_{13}N_5O_3$	O2'-4a-cyclic-tetrahydrobiopterin	Sesquiterpenoids	0.887	0.042
188.0559	pos	1.988	M+H	$C_7H_9NO_5$	1-(Malonylamino)cyclopropanecarboxylic acid	Amino acids, peptides, and analogues	0.912	0.001
204.1239	pos	1.173	M+H	$C_9H_{17}NO_4$	Acetylcarnitine	Fatty acid esters	1.095	0.004
122.0719	pos	0.979	M+H-H$_2$O	$C_6H_9N_3O$	Histidinal	Amines	0.909	0.020
519.331	pos	6.743	M+Na	$C_{24}H_{51}NO_7P+$	1-palmitoylglycerophosphocholine	Glycerophosphocholines	0.947	0.002
148.0611	pos	1.229	M+H	$C_5H_9NO_4$	L-Glutamate	Amino acids, peptides, and analogues	1.053	0.010

Supplementary Table S3 Identification of the most discriminant metabolomic variables between PC group and IR group

**m/z**	**Mode**	**Retention time**	**Adducts**	**Formula**	**Metabolite**	**Subclass**	**Fold change (PC/Control)**	**P value**
227.0663	neg	2.628	M-H, M+FA-H, M+Cl	$C_9H_{12}N_2O_5$	2'-Deoxyuridine	Pyrimidine 2'-deoxyribonucleosides	1.141	0.011
296.6158	neg	6.172	M-2H, M-H, M+Na-2H	$C_{26}H_{45}NO_{10}S_2$	Taurocholic acid 3-sulfate	Bile acids, alcohols and derivatives	0.928	0.050
461.1074	neg	6.152	M-H, 2M-H, M+Na-2H	$C_{22}H_{22}O_{11}$	6-{3,5-dihydroxy-4-[3-(4-methoxyphenyl)prop-2-enoyl]phenoxy}-3,4,5-trihydroxyoxane-2-carboxylic acid	Flavonoid glycosides	1.246	0.000
357.0815	neg	2.957	M-H, M+Na-2H, 2M-H	$C_{15}H_{18}O_{10}$	6-{[3-(2,4-dihydroxyphenyl)propanoyl]oxy}-3,4,5-trihydroxyoxane-2-carboxylic acid	Carbohydrates and carbohydrate conjugates	0.982	0.049
265.1436	neg	6.439	M-H, M+Na-2H	$C_{13}H_{24}O_4$	1,11-Undecanedicarboxylic acid	Fatty acids and conjugates	1.019	0.038
472.1567	neg	2.971	M-H	$C_{20}H_{23}N_7O_7$	Folinic acid	Pterins and derivatives	0.903	0.006
345.063	neg	4.798	M-H	$C_{14}H_{18}O_8S$	3-hydroxy-3-[4-hydroxy-3-(3-methylbut-2-en-1-yl)phenyl]-2-(sulfooxy)propanoic acid	_	1.357	0.000
306.1184	neg	5.645	M+FA-H	$C_{11}H_{19}NO_6$	Epidermin	Carbohydrates and carbohydrate conjugates	1.378	0.029
331.0846	neg	5.994	M+FA-H	$C_{13}H_{18}O_5S$	{[1-(4-methoxyphenyl)-4-methylpent-1-en-3-yl]oxy}sulfonic acid	Anisoles	1.123	0.044

231.0322	neg	6.138	M-H	$C_9H_{12}O_5S$	(4-ethyl-2-methoxyphenyl)oxidanesulfonic acid	Arylsulfates	1.182	0.002
271.1906	neg	6.542	M+FA-H	$C_{14}H_{26}O_2$	Myristoleic acid	Fatty acids and conjugates	1.040	0.029
478.2921	neg	6.789	M-H	$C_{23}H_{46}NO_7P$	PE(18:1(9Z)/0:0)	-	0.830	0.013
271.227	neg	6.878	M-H	$C_{16}H_{32}O_3$	2-hydroxyhexadecanoic acid	Fatty acids and conjugates	1.073	0.019
299.2581	neg	7.002	M-H	$C_{18}H_{36}O_3$	DL-2-hydroxy stearic acid	-	1.072	0.005
327.2893	neg	7.139	M-H	$C_{20}H_{40}O_3$	12-hydroxyicosanoic acid	Fatty acids and conjugates	1.181	0.002
355.3206	neg	7.291	M-H	$C_{22}H_{44}O_3$	2(R)-hydroxydocosanoic acid	Fatty acids and conjugates	1.209	0.031
436.2817	neg	6.844	M-H	$C_{21}H_{44}NO_6P$	PE(P-16:0e/0:0)	-	1.066	0.034
452.2767	neg	6.782	M-H	$C_{21}H_{44}NO_7P$	PE(16:0/0:0)	Glycerophosphoethanolamines	0.831	0.007
283.0603	neg	6.412	M-H	$C_{16}H_{12}O_5$	Acacetin	O-methylated flavonoids	1.287	0.001
309.1333	neg	6.240	M+FA-H	$C_{15}H_{20}O_4$	Tavulin	Terpene lactones	0.891	0.018
379.2112	neg	6.172	M+FA-H	$C_{20}H_{30}O_4$	Prostaglandin B2	Eicosanoids	0.948	0.039
359.0763	neg	6.104	M+FA-H	$C_{17}H_{14}O_6$	2-(3,5-dimethoxyphenyl)-5,7-dihydroxy-4H-chromen-4-one	O-methylated flavonoids	0.811	0.003
263.1279	neg	5.816	M-H	$C_{15}H_{20}O_4$	(+/-)Abscisic Acid	-	0.931	0.040
383.1268	neg	5.563	M+K-2H	$C_{20}H_{26}O_5$	19-Noraldosterone	Hydroxysteroids	0.943	0.029
403.1595	neg	5.193	2M-H	$C_9H_{14}O_5$	3-(1-Hydroxymethyl-1-propenyl)pentanedioic acid	Fatty acids and conjugates	1.030	0.023
303.0531	neg	4.825	M+FA-H	$C_{11}H_{14}O_5S$	(3-methyl-2-oxo-4-phenylbutoxy)sulfonic acid	Phenylpropanes	1.099	0.018
217.107	neg	4.627	M+FA-H	$C_9H_{16}O_3$	Cis-3-Hexenyl lactate	Carboxylic acid derivatives	1.024	0.030
317.0324	neg	3.366	M+FA-H	$C_{11}H_{12}O_6S$	[(5-hydroxy-2-methyl-2H-chromen-2-yl)methoxy]sulfonic acid	1-benzopyrans	1.155	0.025
361.1129	neg	3.142	M+FA-H	$C_{14}H_{20}O_8$	Vanilloloside	Carbohydrates and carbohydrate conjugates	0.959	0.013

225.087	neg	2.882	M-H	$C_{10}H_{14}N_2O_4$	Porphobilinogen	Amines	0.980	0.014
155.0084	neg	1.139	M-H	$C_5H_4N_2O_4$	Orotic acid	Pyrimidines and pyrimidine derivatives	0.960	0.019
463.1253	pos	6.148	M+H, M+Na, $M+NH_4$, $M+H-H_2O$	$C_{22}H_{22}O_{11}$	3,4,5-trihydroxy-6-[4-(7-hydroxy-8-methoxy-4-oxo-3,4-dihydro-2H-1-benzopyran-2-yl)phenoxy]oxane-2-carboxylic acid	Flavonoid glycosides	1.331	0.000
239.0923	pos	3.680	M+H, M+ACN+Na	$C_{12}H_{14}O_5$	3,4,5-Trimethoxycinnamic acid	Hydroxycinnamic acids and derivatives	1.022	0.049
299.0928	pos	4.858	$M+H-H_2O$, M+ACN+H	$C_{17}H_{16}O_6$	6-hydroxy-2-(4-hydroxyphenyl)-7,8-dimethoxy-3,4-dihydro-2H-1-benzopyran-4-one	O-methylated flavonoids	1.248	0.000
482.3622	pos	7.037	M+H, M+Na	$C_{24}H_{52}NO_6P$	PC(O-16:0/0:0)	-	1.046	0.026
136.0624	pos	1.166	M+H, $2M+NH_4$	$C_5H_5N_5$	Adenine	Purines and purine derivatives	1.054	0.037
317.0881	pos	3.026	$M+H-2H_2O$, M+H	$C_{13}H_{16}O_9$	3,4,5-trihydroxy-6-(2-hydroxy-6-methoxyphenoxy)oxane-2-carboxylic acid	Carbohydrates and carbohydrate conjugates	1.421	0.000
144.0484	pos	0.993	M+H	C_6H_9NOS	4-Methyl-5-thiazoleethanol	Thiazoles	0.958	0.013
132.0774	pos	1.007	M+H	$C_4H_9N_3O_2$	Creatine	Amino acids, peptides, and analogues	1.065	0.013
140.0348	pos	2.678	$M+H-2H_2O$	$C_6H_9NO_5$	N-Formyl-L-glutamic acid	Amino acids, peptides, and analogues	0.970	0.027
137.0967	pos	4.543	M+H	$C_9H_{12}O$	P-Isopropylphenol	Cumenes	1.029	0.010
165.0918	pos	4.550	M+H	$C_{10}H_{12}O_2$	THYMOQUINONE	Carbonyl compounds	1.035	0.009
201.113	pos	4.620	M+H	$C_{10}H_{16}O_4$	Decenedioic acid	Fatty acids and conjugates	1.026	0.034

176.1078	pos	5.534	M+H	$C_{11}H_{13}NO$	N-Acetyltranylcypromine	-	0.929	0.036
370.1671	pos	6.093	M+NH4	$C_{21}H_{20}O_5$	Artonol A	1-benzopyrans	0.834	0.000
300.218	pos	6.100	M+H-H2O	$C_{16}H_{31}NO_5$	3-hydroxynonanoyl carnitine	Fatty acid esters	0.962	0.018
285.0768	pos	6.406	M+H	$C_{16}H_{12}O_5$	Genkwanin	-	1.220	0.002
119.0863	pos	6.455	M+H	C_9H_{10}	Alpha-Methylstyrene	Phenylpropenes	0.977	0.028
500.2615	pos	6.638	M+ACN+H	$C_{26}H_{34}O_7$	6-hydroxy-5-{[(3-hydroxy-2-oxo-2H-chromen-7-yl)oxy]methyl}-1,1,4a,6-tetramethyl-decahydronaphthalen-2-yl acetate	Hydroxycoumarins	0.928	0.035
497.2738	pos	6.518	M+CH3OH+H	$C_{25}H_{36}O_8$	Testosterone glucuronide	Steroidal glycosides	1.229	0.009
460.2711	pos	6.462	M+NH4	$C_{26}H_{34}O_6$	3-O-Acetylepisamarcandin	_	0.975	0.015
474.2869	pos	6.455	M+NH4	$C_{27}H_{36}O_6$	Lucidenolactone	Triterpenoids	0.961	0.036
135.0811	pos	6.455	M+H	$C_9H_{10}O$	2,5-Dimethylbenzaldehyde	Benzoyl derivatives	0.978	0.045
398.2916	pos	6.197	M+H	$C_{22}H_{39}NO_5$	AMP-Deoxynojirimycin	-	0.963	0.025
414.1569	pos	6.128	M+ACN+H	$C_{20}H_{20}O_7$	6-(2,4-dihydroxyphenyl)-2-(2,6-dihydroxyphenyl)-5-hydroxy-4-methylcyclohex-3-ene-1-carboxylic acid	Benzenediols	0.851	0.001
99.04497	pos	5.639	M+H	$C_5H_6O_2$	2-Furanmethanol	_	1.053	0.048
299.0929	pos	5.437	M+H-H2O	$C_{17}H_{16}O_6$	5,7-dihydroxy-6-methoxy-2-(4-methoxyphenyl)-3,4-dihydro-2H-1-benzopyran-4-one	O-methylated flavonoids	0.912	0.018
139.076	pos	5.018	M+H	$C_8H_{10}O_2$	2-(4-Hydroxyphenyl)ethanol	Tyrosols and derivatives	1.023	0.018
372.2418	pos	4.342	2M+3H2O+2H	$C_{15}H_{30}N_6O_4$	Kinetensin 1-3	Amino acids, peptides, and analogues	1.151	0.002

305.0778	pos	3.444	M+ACN+H	$C_9H_{13}NO_6S$	N-acetyl-S-(3-oxo-3-carboxy-n-propyl)cysteine	Amino acids, peptides, and analogues	0.917	0.036
276.0699	pos	3.082	M+H	$C_{10}H_{13}NO_8$	3-beta-D-Glucopyranuronosyloxy-5-methylisoxazole	-	0.704	0.013
456.2106	pos	2.790	M+H-H_2O	$C_{25}H_{27}N_7O_3$	CD 1790	Purines and purine derivatives	0.966	0.002
113.0354	pos	2.629	M+H	$C_4H_4N_2O_2$	Uracil	Pyrimidines and pyrimidine derivatives	1.115	0.007
248.1502	pos	2.197	M+H	$C_{11}H_{21}NO_5$	(R)-3-hydroxybutyrylcarnitine	Fatty acid esters	0.968	0.040
188.0559	pos	1.988	M+H	$C_7H_9NO_5$	1-(Malonylamino)cyclopropanecarboxylic acid	Amino acids, peptides, and analogues	0.947	0.002
365.1445	pos	1.647	M+ACN+Na	$C_{17}H_{19}NO_4$	Oxymorphone	_	0.978	0.015
112.0878	pos	0.701	M+H	$C_5H_9N_3$	Histamine	Amines	0.966	0.004
93.0376	pos	0.603	M+H	C_3H_8OS	2-(Methylthio)ethanol	Dialkylthioethers	0.980	0.005
115.0873	pos	0.979	M+H-H_2O	$C_5H_{12}N_2O_2$	Ornithine	Amino acids, peptides, and analogues	0.983	0.001
